# Pyrrolidine nucleotide analogs with a tunable conformation

**DOI:** 10.3762/bjoc.10.205

**Published:** 2014-08-22

**Authors:** Lenka Poštová Slavětínská, Dominik Rejman, Radek Pohl

**Affiliations:** 1 Institute of Organic Chemistry and Biochemistry, Academy of Sciences of the Czech Republic, Flemingovo nám. 2, 166 10 Prague 6, Czech Republic

**Keywords:** conformation, NMR, nucleic acids, nucleotide analog, phosphonic acid, pseudorotation, pyrrolidine

## Abstract

Conformational preferences of the pyrrolidine ring in nucleotide analogs **7–14** were investigated by means of NMR and molecular modeling. The effect of the relative configuration of hydroxy and nucleobase substituents as well as the effect of the alkylation or acylation of the pyrrolidine nitrogen atom on the conformation of the pyrrolidine ring were studied. The results of a conformational analysis show that the alkylation/acylation can be effectively used for tuning the pyrrolidine conformation over the whole pseudorotation cycle.

## Introduction

**Figure 1:**
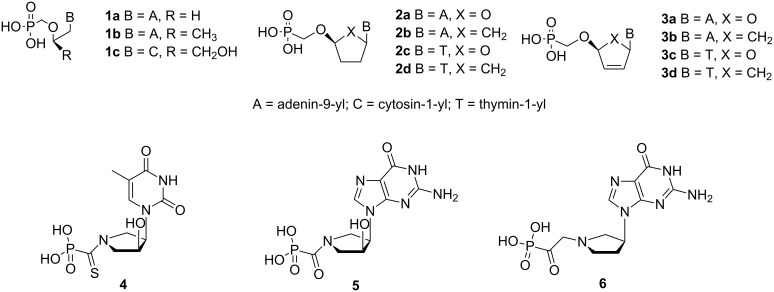
Examples of biologically active acyclic and cyclic nucleotide analogs.

Nucleotides, nucleosides and nucleobases play an important role in all biological systems. Therefore, it is not surprising that many of their analogs possess interesting biological properties. Potent antiviral drugs based on phosphonate nucleotides **1a–c** [1–2], **2a–d** and **3a–d** (Figure 1) have been reported. Prodrugs of acyclic compounds **1a–c** are currently in clinical use for the treatment of diseases caused by DNA viruses and retroviruses. Cyclic analogs **2** and **3** were reported to exhibit antiviral activity against HIV strains [3]. These examples show that the modification of the sugar-phosphate moiety in nucleotides is a successful approach in developing antiviral therapeutics.

**Figure 2:**
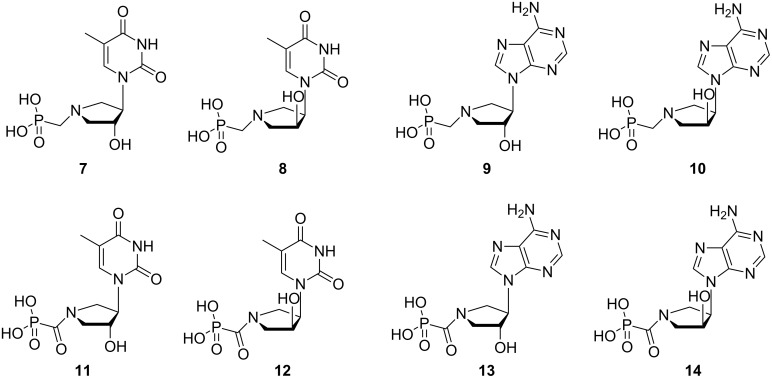
The pyrrolidine nucleotide analogs investigated in this study.

Our long-term interest in the synthesis and evaluation of biological properties of phosphonate azanucleotides has yielded several potent inhibitors of nucleoside/nucleotide metabolizing enzymes: thymine derivatives **4** and **11** (Figure 1 and Figure 2) – inhibitors of thymidine phosphorylase isolated from spontaneous lymphoma of SD rats (IC_50_ = 15 and 11 nM, respectively) [4], guanine derivative **5** – a potent inhibitor of human purine nucleoside phosphorylase PNP (*K*_i_ = 10 nM) [5], and finally guanine derivative **6** – exhibiting inhibitory activity against 6-oxopurine phosphoribosyltransferases from *Escherichia coli* [6]. These biologically active analogs contain a five-membered pyrrolidine ring, whose conformation has not been explored so far.

It is known that the conformation of a five-membered ribose or deoxyribose ring plays an important role in the nucleoside, nucleotide and oligonucleotide spatial structure. The conformation predefines, for example, the stability of DNA:RNA duplexes – complexes between oligodeoxyribonucleotide and a complementary RNA strain [7] or an overall shape of oligonucleotide. It is also well known that the structure of DNA may alternate between A, B and Z forms depending on the hydration and the type and concentration of metal ions, which is also reflected in the conformation of the sugar part. Finally, the conformation of ribose or its mimics also significantly predetermines the binding of a nucleoside, nucleotide or an analog thereof to an active site of a particular enzyme. The knowledge of the conformation of nucleosides, nucleotides and their analogs is therefore essential for the understanding or even prediction of their biological properties.

There are several approaches providing information on the conformation of a five-membered ring. Conformational analysis using vicinal proton–proton scalar couplings (^3^*J*_HH_) of ring protons obtained from ^1^H NMR spectra is a traditional and well-established experimental method. ^3^*J*_HH_ encodes information on the exocyclic dihedral angle between coupled protons based on the Karplus relationship [8]. Exocyclic dihedral angles are in turn in direct relation with endocyclic dihedral angles between ring atoms defining the conformation of the five-membered ring based on the pseudorotation concept [9]. This straightforward approach is complicated by the presence of more than one rapidly interconverting conformer in solution, resulting in the observation of averaged experimental ^3^*J*_HH_. Nevertheless, even these averaged ^3^*J*_HH_ can be used for conformational analysis by means of the program PSEUROT [10], which minimizes differences between experimental and calculated ^3^*J*_HH_, assuming a two-state equilibrium of conformers. The output of the PSEUROT provides pseudorotation parameters: phase angles (*P*), the maximum puckering amplitudes (Φ_max_) and the relative amounts of individual conformers. The original PSEUROT program was later complemented with the program MULDER [11]. Recently, the Matlab Pseudorotation GUI version of the program has been developed, enabling the creation of conformational maps [12].

The conformational analysis using PSEUROT-based programs was originally designed for ribose or deoxyribose, but in general it can be used for any saturated five-membered ring. The program PSEUROT was, for example, used in a conformational analysis of the prolyl ring in aminoethylprolyl peptide nucleic acid monomers [13], or in a conformational analysis of 2’-fluoro-4’-thioarabinothymidine [14]. The performance of the Matlab Pseudorotation GUI program was tested on two 4’-thio-2’deoxynucleoside analogs [12].

In this publication, we present a conformational analysis of pyrrolidine azanucleotide analogs **7–14** containing thymine and adenine as examples of pyrimidine and purine nucleobases, respectively (Figure 2), and show how the conformation is affected by the mode of the phosphonate moiety attachment to the pyrrolidine ring. Particularly, we show that the alkylation or acylation (an amine or an amide bond formation) of the pyrrolidine nitrogen atom can be used for a simple but effective tuning of pyrrolidine-ring conformation in compounds **7–14**.

## Results and Discussion

### Chemistry

**Scheme 1:**
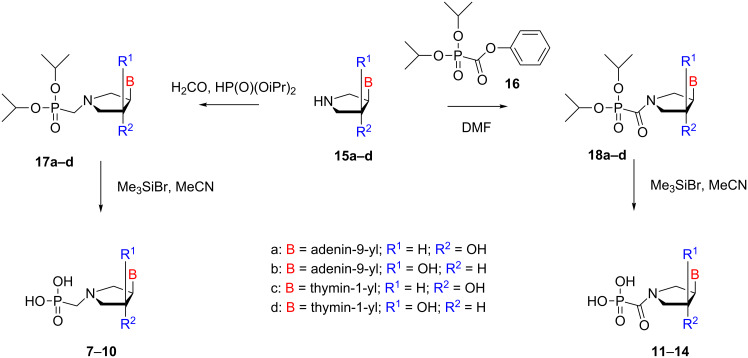
The synthesis of pyrrolidine nucleotides **7**–**14**.

Phosphonomethyl derivatives **7–10** were prepared from pyrrolidine azanucleosides **15a–d** [5,15] via a Mannich-type reaction with diisopropyl phosphite and aqueous formaldehyde at elevated temperature in good yields (Scheme 1). The obtained diisopropyl esters **17a–d** were deprotected by treatment with trimethylsilyl bromide in acetonitrile at room temperature.

Phosphonoformyl derivatives **11–14** were prepared from **15a–d** by the reaction with diisopropyl phenylphosphonoformate (**16**) at elevated temperature in good yields. The obtained diisopropyl esters **18a–d** were deprotected by treatment with trimethylsilyl bromide in acetonitrile at room temperature.

### NMR and conformational analysis

**Figure 3:**
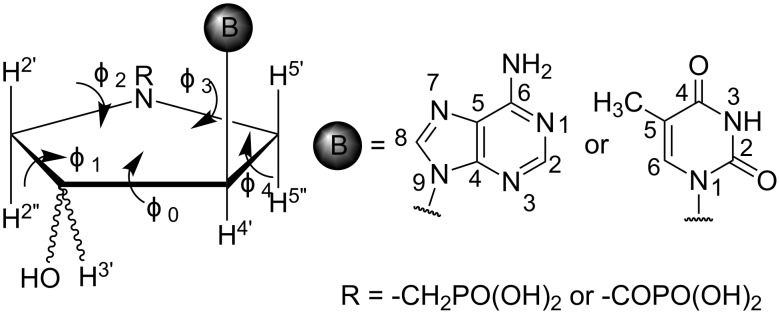
The numbering of the pyrrolidine ring, the nucleobase and the endocyclic phase angles for the purposes of the conformational analysis.

Phosphonomethyl **7–10** and phosphonoformyl **11–14** analogs were characterized by ^1^H, ^13^C and ^31^P NMR. The NMR spectra were acquired using 50 mM solutions of the studied compounds in D_2_O and all resonances were assigned based on H,H-COSY, H,H-ROESY, H,C-HSQC and H,C-HMBC experiments (for a complete signal assignment and copies of NMR spectra, see Supporting Information File 1). The numbering of the pyrrolidine ring, the nucleobase and the endocyclic phase angles Φ_0_–Φ_4_ for the purposes of the conformational analysis is shown in Figure 3. There are fundamental differences in the NMR spectra of phosphonomethyl and phosphonoformyl derivatives, which will be commented on in detail in the following paragraphs.

### NMR study of phosphonomethyl derivatives

**Figure 4:**
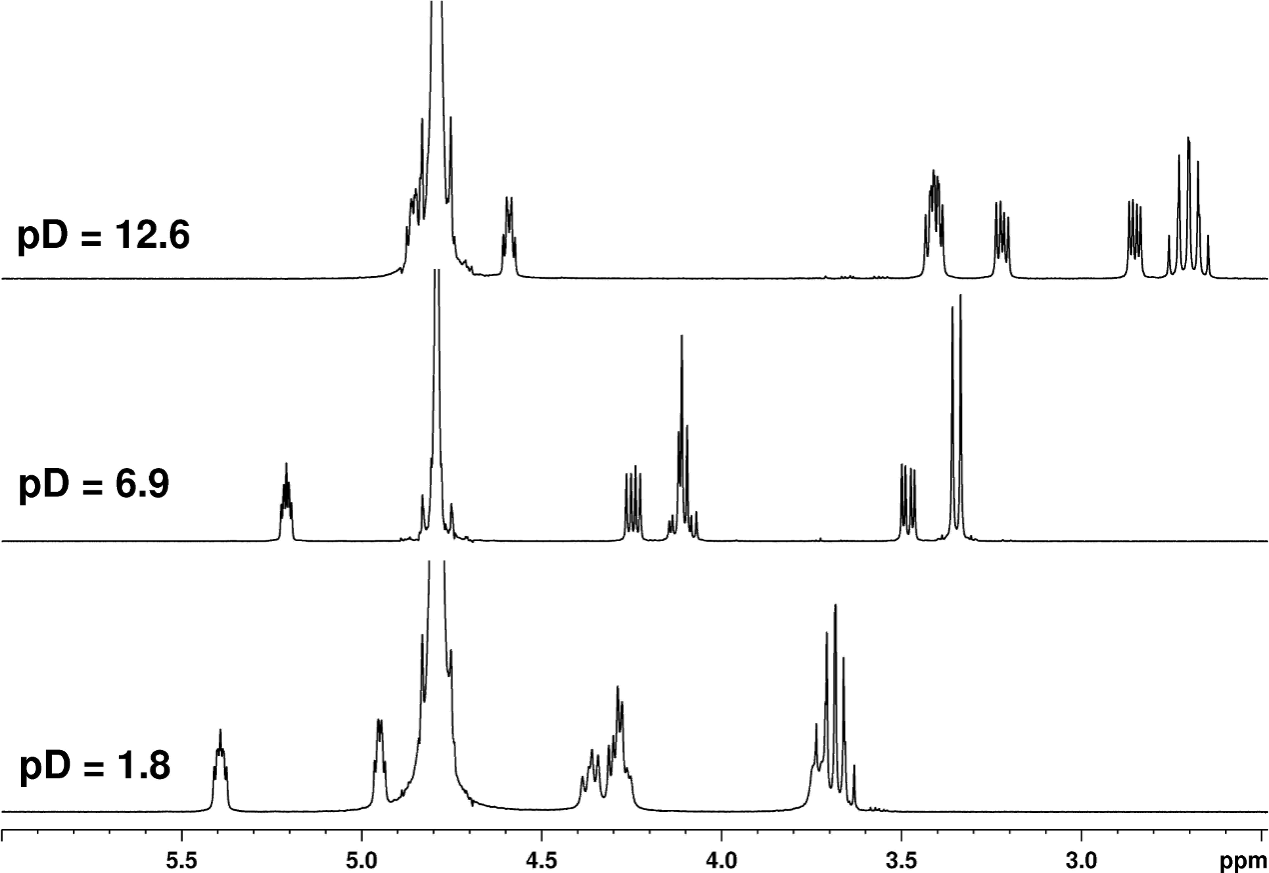
The aliphatic part (pyrrolidine protons) of the ^1^H NMR spectra of **9** measured in D_2_O at different pD values.

Phosphonomethyl derivatives **7–10** contain both basic (pyrrolidine) and acidic (phosphonic acid) functionalities. Therefore, several protonation/deprotonation transitions are expected to take place when scanning a pH scale. In order to determine these transitions, we measured NMR spectra of **7–10** at different pD values using D_2_O solutions of DCl or NaOD for decreasing or increasing pD, respectively. We found that the NMR spectra of **7–10** are strongly pD-dependent as demonstrated for example on the ^1^H NMR spectrum of adenine derivative **9** (Figure 4).

**Figure 5:**
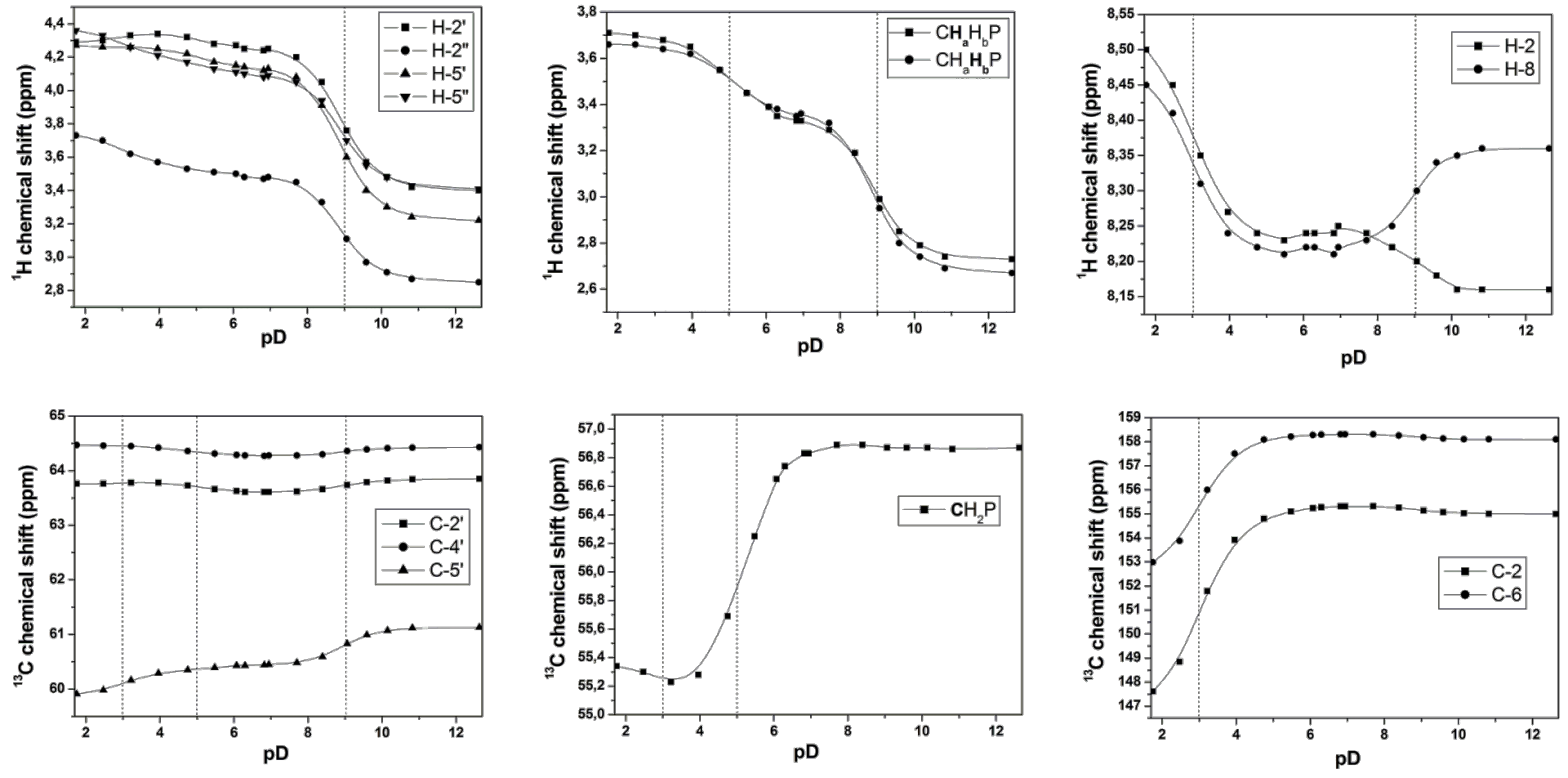
Changes of selected ^1^H and ^13^C chemical shifts of **9** upon pD change.

**Figure 6:**
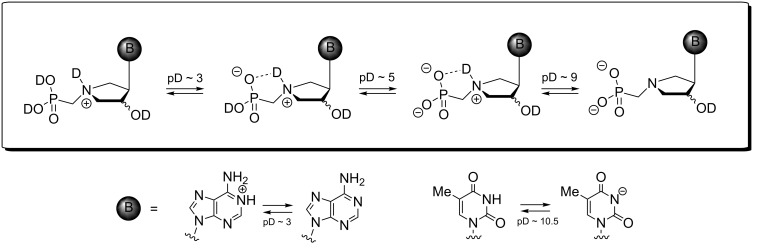
The deuteration equilibria of phosphonomethyl derivatives **7–10**.

As follows from pD titrations (Figure 5; for more details see Supporting Information File 1), phosphonomethyl analogs **7–10**, both *cis* (**8**, **10**) and *trans* (**7**, **9**), exist at a very low pD value (<3) as free phosphonic acids with a deuterated pyrrolidine nitrogen atom (Figure 6). The phosphonic acid moiety goes through a two-stage deuteration/dedeuteration transition at pD ~ 3 and 5 and the pyrrolidine nitrogen atom stays deuterated until pD ~ 9. This suggests that the compounds exist as zwitterions with a hydrogen bond between the negatively charged phosphonate moiety and the positively charged pyrrolidine nitrogen atom in the range of pD 3–9. The pD titration experiments have also revealed the deuteration/dedeuteration of adenine and thymine nucleobases. In agreement with the data reported [16–17], adenine becomes deuterated predominantly on N-1 at pD ~ 3 while thymine releases its deuterium at N-3 at pD ~ 10.5 and becomes negatively charged. Typically, a pD value of 5.7–6.9 was reached when 50 mM solutions of **7–10** in D_2_O were prepared for the NMR measurement, which indicates that isolated compounds are in the form of the monosodium monozwitterionic salt.

The orientation of the phosphonomethyl moiety relative to the nucleobase in the zwitterionic form could be theoretically deduced from the H,H-ROESY spectra. In real spectra, however, protons from C*H_2_*PO(OH)_2_ provide NOE contacts to protons on both sides of the pyrrolidine ring, suggesting an exchange of deuterium connected to the pyrrolidine nitrogen.

### NMR study of phosphonoformyl derivatives

**Figure 7:**
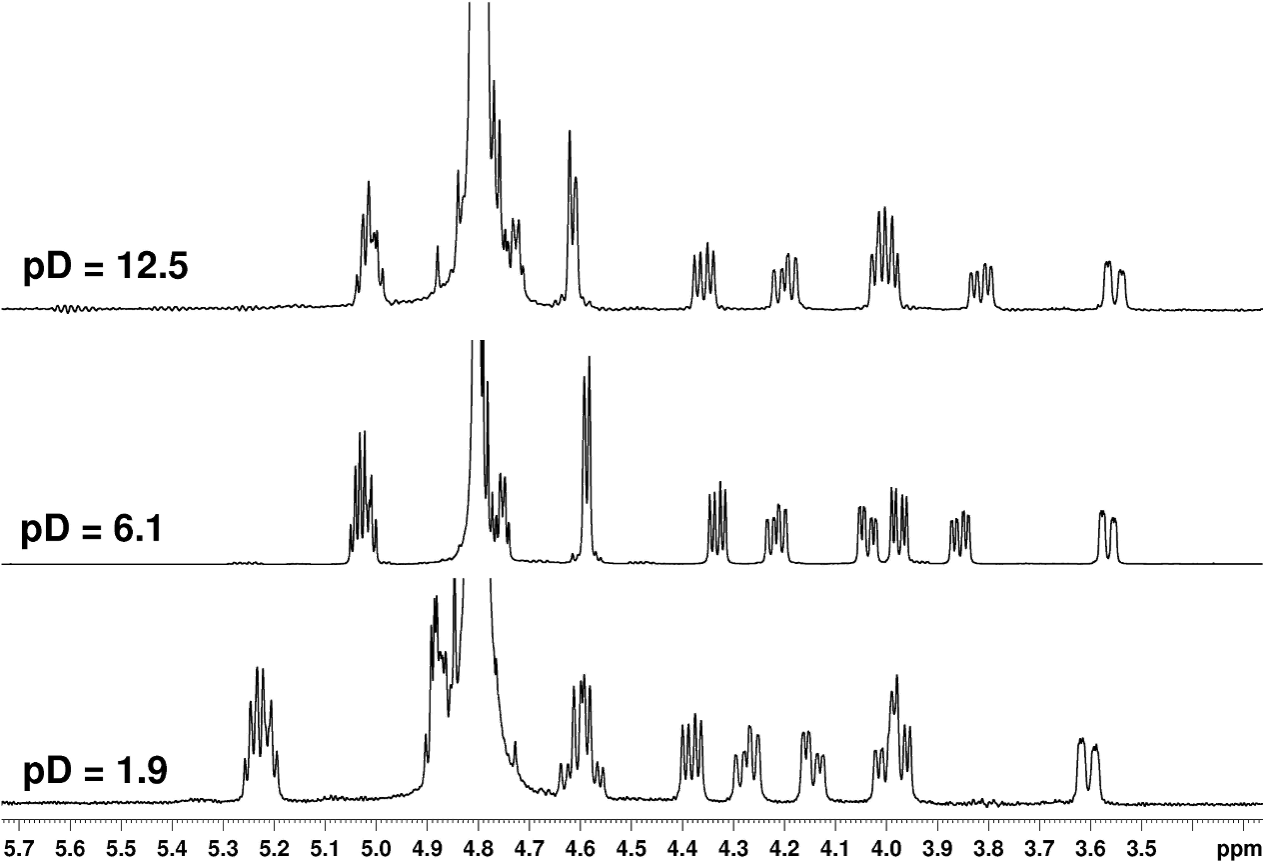
The aliphatic part (pyrrolidine protons) of the ^1^H NMR spectra of **13** measured in D_2_O at different pD values.

In contrast to **7–10**, an amidic pyrrolidine nitrogen atom in phosphonoformyl derivatives **11–14** is not involved in protonation/deprotonation transitions. Only negligible changes of ^1^H chemical shifts were observed when the pD value of the solution was changed as demonstrated in the example of compound **13** (Figure 7).

**Figure 8:**
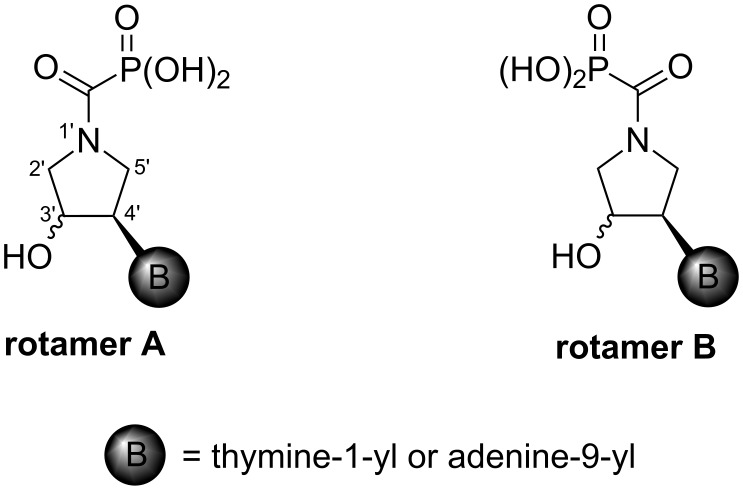
Amide rotamers of phosphonoformyl derivatives **11–14**.

Indeed, the reason is the delocalization of the lone electron pair on the pyrrolidine nitrogen atom by resonance. This brings, in addition to the lack of basicity, the existence of two amide rotamers A and B (Figure 8), which can be observed in NMR spectra as two sets of signals. The ratio of A:B is about 1:1 with a slight excess of rotamer B (see the Experimental and Supporting Information File 1).

**Figure 9:**
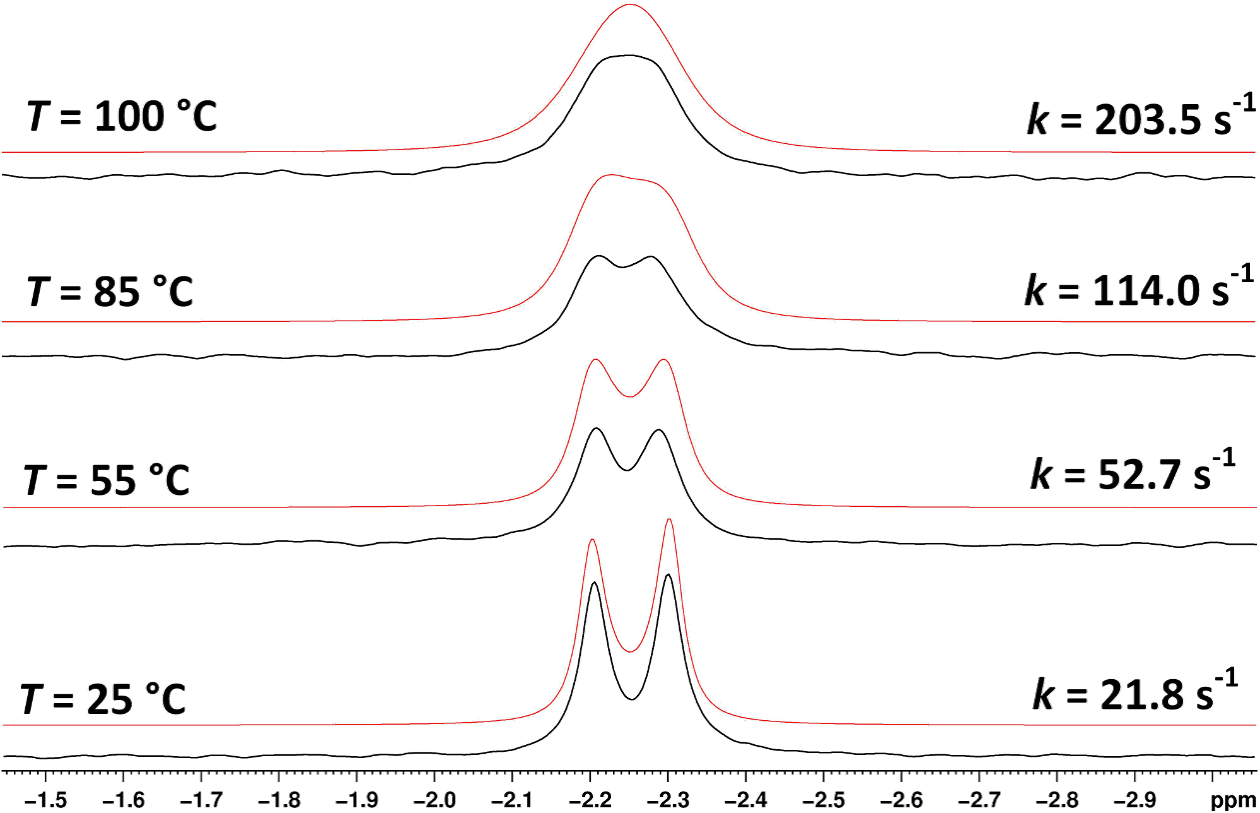
The ^31^P NMR spectra (202.3 MHz) of **14** measured (the black curve) and simulated (the red curve) at various temperatures.

The existence of the two rotamers prompted us to estimate the energy barrier of their interconversion. We therefore performed variable ^31^P NMR measurements of **14** at different temperatures (starting at 25 °C with a 10 °C step, with the last measurement taken at 100 °C) and a line-shape analysis of an uncoupled, exchanging two-site ^31^P system (Figure 9). This enabled us to obtain the rate constants of the exchange at different temperatures and estimate the activation parameters of the interconversion such as the Gibbs free energy of activation Δ*G*^‡^_298_ = 80.7 kJ/mol; the enthalpy of activation Δ*H*^‡^ = 23.8 kJ/mol; and the entropy of activation Δ*S*^‡^ = −190.6 J/(K·mol) (for details, see Supporting Information File 1). The relatively low value of Δ*G*^‡^_298_ = 80.7 kJ/mol implies that the preparative isolation of the individual rotamers A and B at room temperature would not be possible.

**Figure 10:**
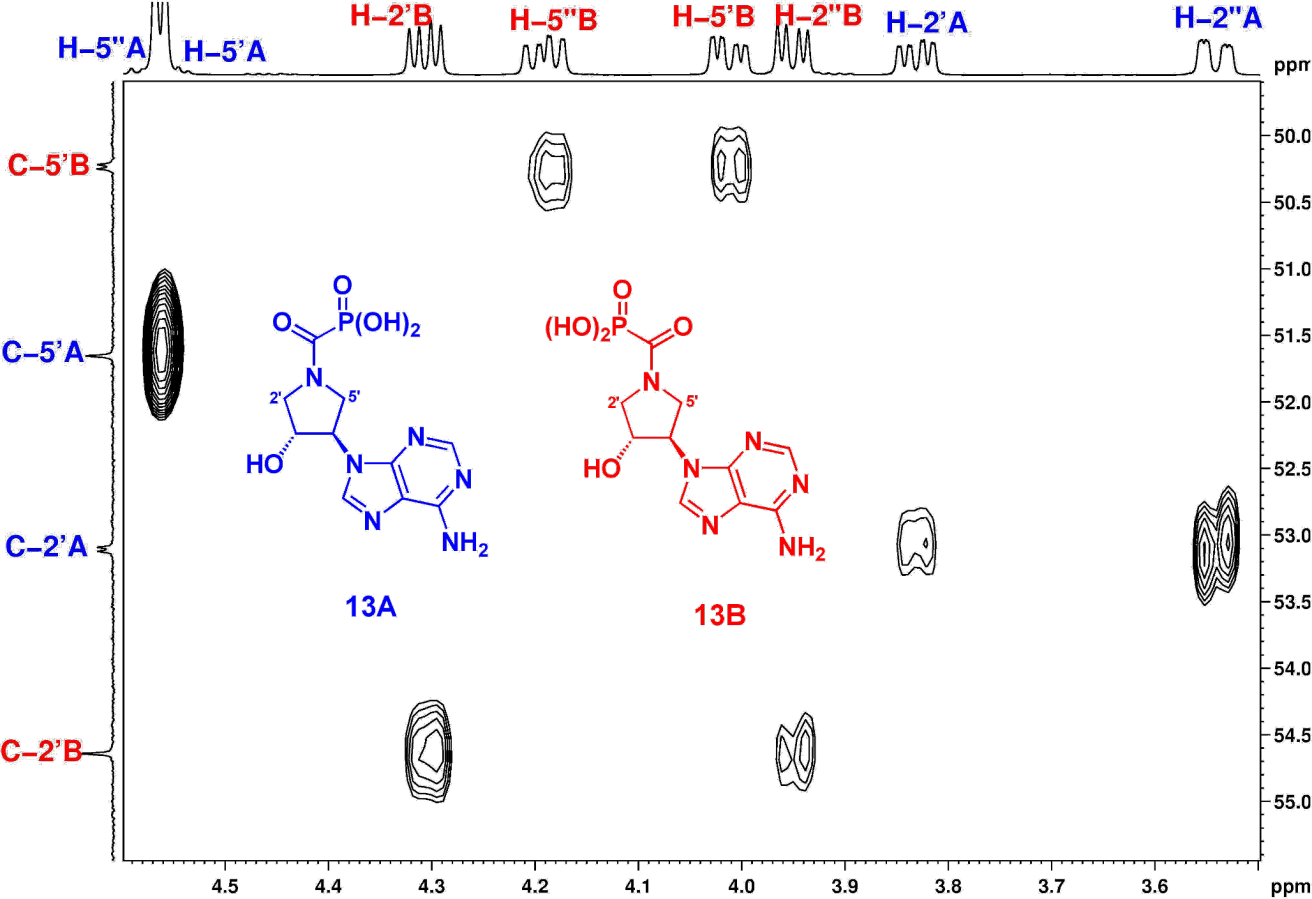
A part of the H,C-HSQC spectrum of derivative **13**, showing the assignment of rotamers A and B.

For the purposes of conformational analysis, it was necessary to assign correctly the NMR signals of the individual conformers. We noticed that only one of the C-2’ or C-5’ signals in the particular rotamer is split in the ^13^C NMR spectrum due to ^3^*J*(C,P) spin–spin interaction. In harmony with the general dependence of the vicinal coupling constant on the dihedral angle according to the well-known Karplus relationship, one would expect the ^3^*J*(C,P) of the carbon in the *trans* arrangement to the phosphorus atom to evince larger splitting than the other arranged in the *cis* arrangement. Thus, ^3^*J*(C2’,P) > ^3^*J*(C5’,P) in rotamer A and ^3^*J*(C5’,P) > ^3^*J*(C2’,P) in rotamer B should be observed. In order to support this assumption, we calculated ^3^*J*(C,P) of the most stable conformers (see the chapter Conformational analysis) of the adenine derivatives **13** and **14** for both A and B rotamers using the DFT B3LYP/6-31++G* method. The results summarized in Table 1 show a good agreement of the calculated and experimental values, confirming correct assignment of the rotamers (Figure 10).

**Table 1:** The comparison of the observed and calculated (in parentheses) ^3^*J*(C2’,P) and ^3^*J*(C5’,P) for rotamers **13A,B** and **14A,B**.

Rotamer	^3^*J*(C2’,P)	^3^*J*(C5’,P)

**13A**	4.6 (3.8)	0 (−0.8)
**13B**	0 (−0.3)	4.9 (4.8)
**14A**	4.5 (4.2)	0 (−0.8)
**14B**	0 (−0.3)	4.9 (4.8)

### Conformational analysis

**Figure 11:**
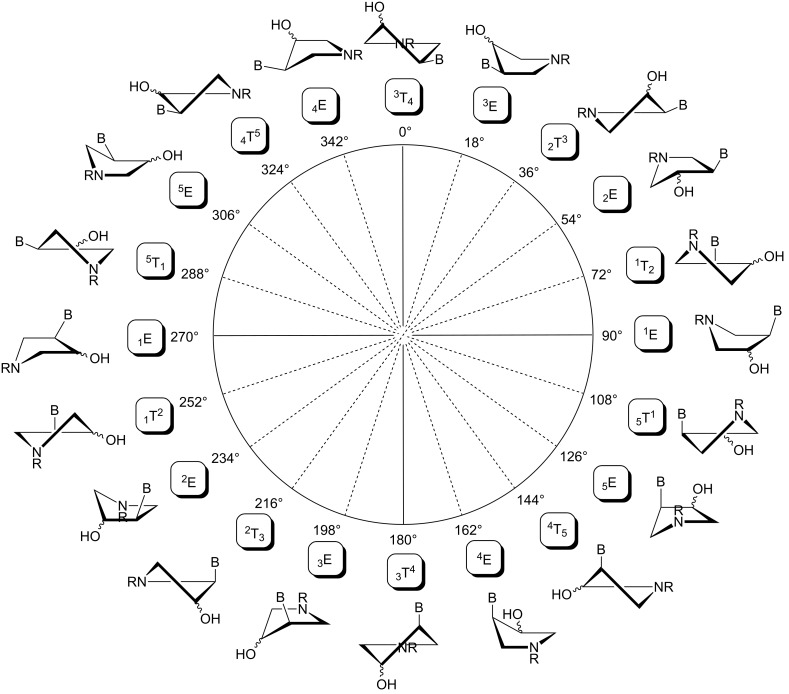
The pseudorotation pathway of the pyrrolidine ring in the compounds studied. The sign B stands for a nucleobase (thymin-1-yl or adenin-9-yl) and R means a phosphonomethyl or phosphonoformyl moiety.

The geometry of the pyrrolidine ring in the compounds studied can occupy various envelope (E) and twisted (T) conformations as depicted in Figure 11. The particular conformation is described by two pseudorotation parameters: by the phase angle (*P*) and by the maximum puckering amplitude (Φ_max_) [9]. The phase angle is a periodic variable indicating which ring atoms are situated outside the ring plane and can reach 0°–360°. The maximum puckering amplitude describes the degree of distortion of the five-membered ring out of the plane and its value is usually in the range of 35°–45°.

Conformational analysis using experimental vicinal ^1^H–^1^H scalar coupling constants (^3^*J*_HH_) together with PSEUROT-based programs is aimed at fitting the calculated ^3^*J*_HH_ to experimental values assuming a two-state conformation equilibrium. In this study, we used the Matlab Pseudorotation GUI program [12] to perform this task. The program requires several input parameters that include experimental ^3^*J*_HH_ and constants A and B, describing the relation between exocyclic Φ_exo_ (related to ^3^*J*_HH_) and endocyclic Φ_endo_ (related to *P* and Φ_max_) dihedral angles (Φ_exo_ = AΦ_endo_ + B).

**Figure 12:**
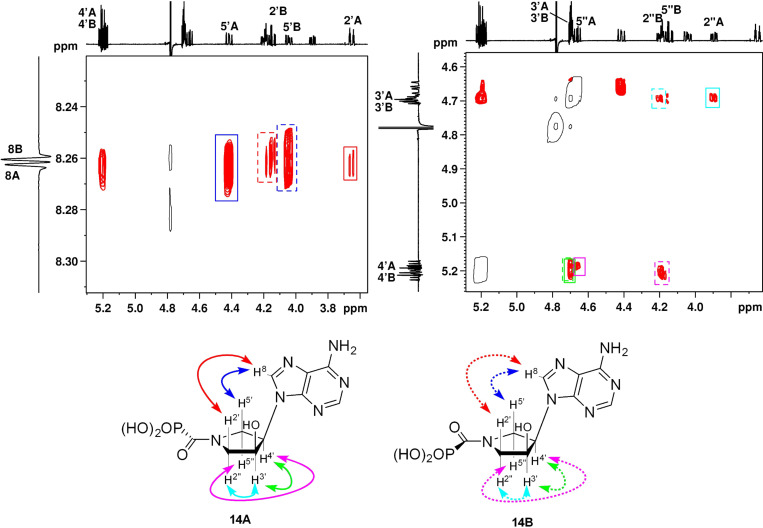
An example of the stereospecific assignment of pyrrolidine-ring protons of **14** in the H,H-ROESY spectrum.

The values of ^3^*J*_HH_ (Table 2) were extracted from the ^1^H NMR spectra, in which the signals were stereospecifically assigned based on ROESY cross-peaks as demonstrated on the example of compound **14** in Figure 12.

**Table 2:** The ^3^*J*_HH_ of the pyrrolidine-ring protons used in the conformational analysis.

	2’,3’	2”,3’	3’,4’	4’,5’	4’,5”

**7**	6.9	6.4	4.1	5.6	9.1
**8**	2.0	5.2	6.0	6.9	9.4
**9**	6.5	5.1	3.0	4.4	7.3
**10**	3.5	6.1	6.2	5.0	7.9
**11A**	6.8	4.8	5.4	6.2	7.7
**11B**	6.1	5.7	5.5	6.1	8.5
**12A**	2.1	5.2	4.5	9.5	8.1
**12B**	3.3	4.8	4.6	8.5	8.5
**13A**	6.1	4.1	4.5	5.6	6.3
**13B**	5.8	5.2	5.3	5.5	7.8
**14A**	2.1	5.1	4.2	9.2	7.6
**14B**	3.5	4.6	4.3	8.3	8.2

The parameters A and B for the five-membered ribose and deoxyribose ring had been traditionally obtained from X-ray data [18]. In the case of a five-membered ring for which X-ray data are not available, DFT-optimized geometries of a number of conformations are used [19]. This molecular modeling approach was also employed in the case of the studied compounds **7–14**. We performed the ring parameterization for adenine derivatives **9**, **10**, **13** and **14** (see Supporting Information File 1 for details) and assumed that replacing adenine for thymine as a nucleobase would not dramatically affect the constants A and B.

The results of the conformational analysis obtained using the Matlab Pseudorotation GUI program are summarized in Table 3. Instead of presenting the pseudorotation parameters *P* and Φ_max_ in numbers, we rather present the pseudorotation maps generated by the program in which the contour plots indicate the root-mean-square-deviation between the fitted and experimental ^3^*J*_HH_. These conformation maps provide a more realistic view of the conformational behavior of compounds **7–14** than a single numeric value for *P* and Φ_max_.

**Table 3:** The results of the conformational analysis.

Analog	Pseudorotation map^a^	Conformer population^b^	Conformation preferences^c^

**7**	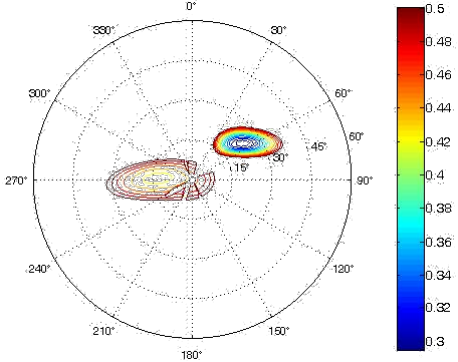	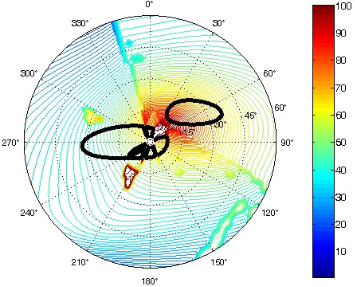	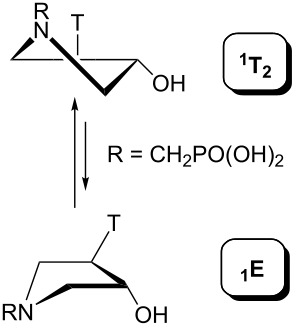
**8**	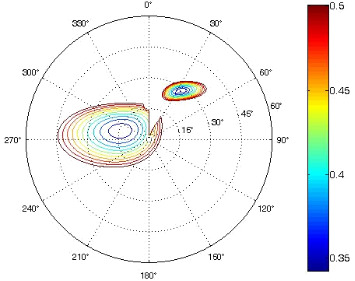	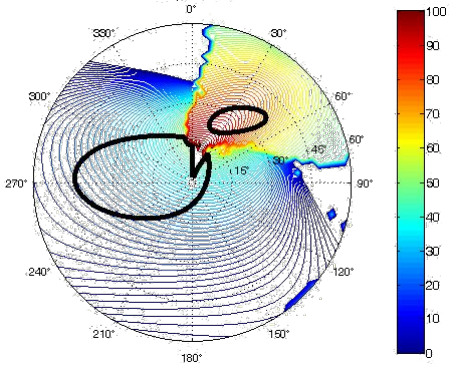	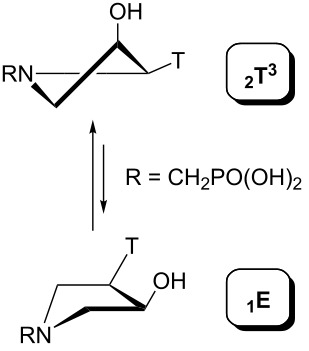
**9**	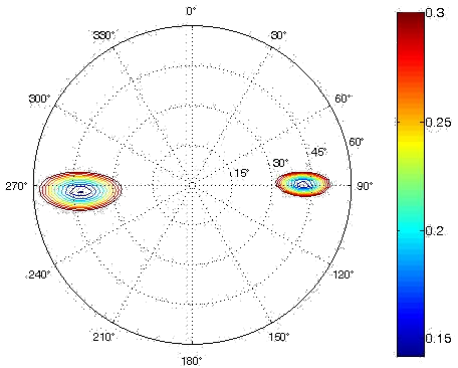	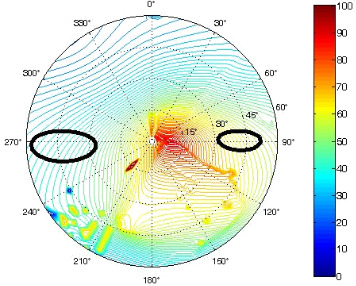	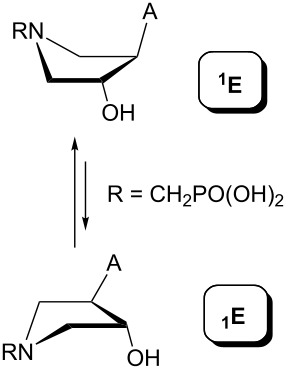
**10**	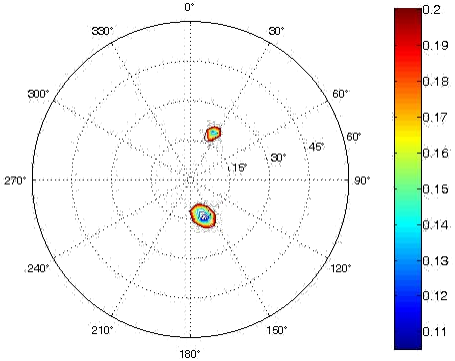	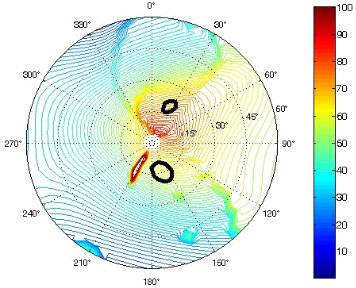	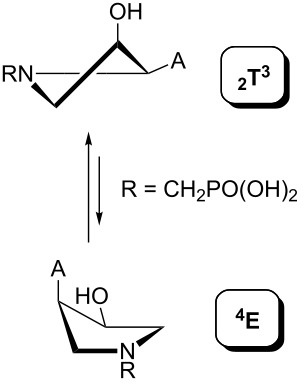
**11A**	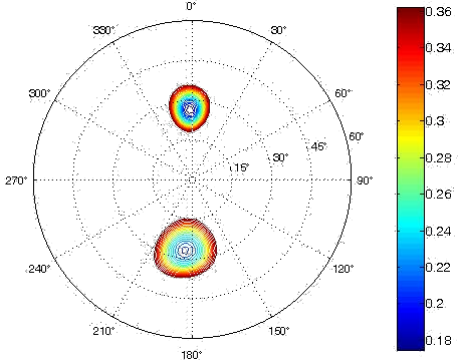	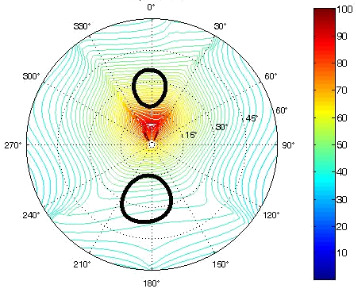	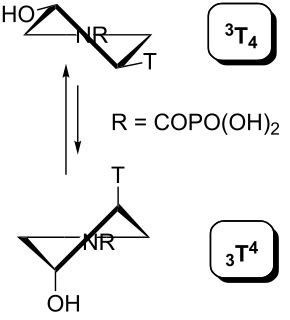
**12A**	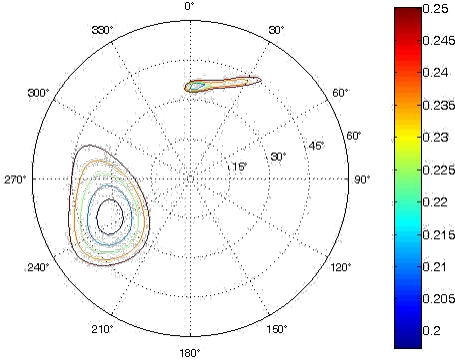	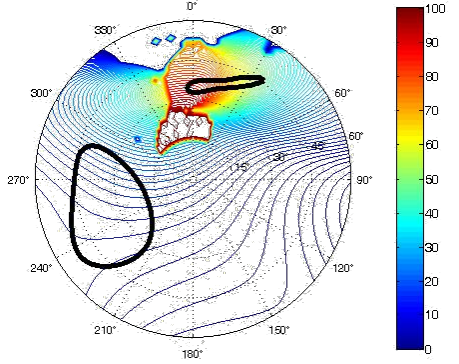	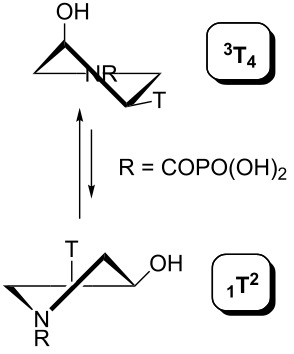
**13A**	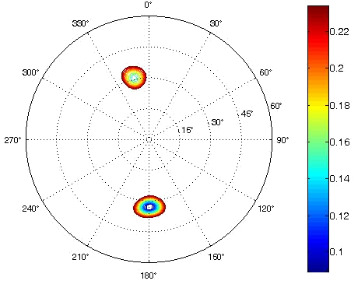	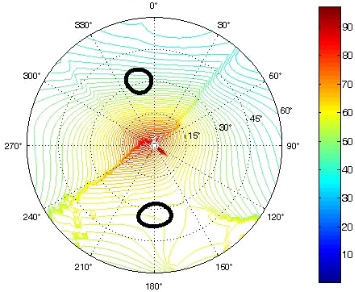	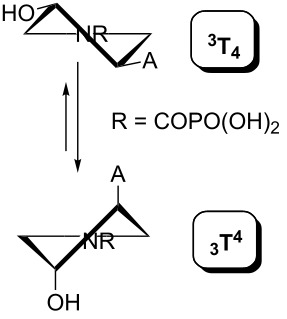
**14A**	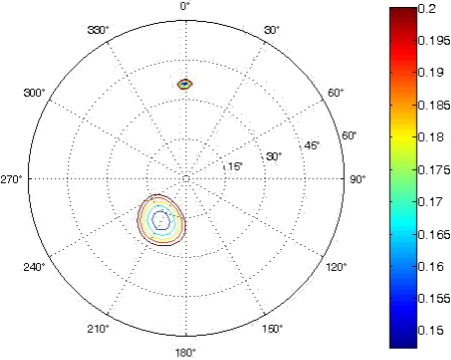	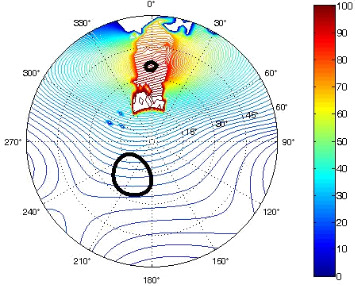	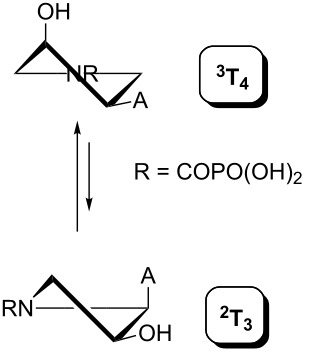

^a^The contour lines indicate the total RMSD of the fit. ^b^The contour lines indicate the percentage of the conformation present in the best fit. The thicker black line is the outer contour line of the two dominant conformations from the second column of the table. ^c^A graphical illustration of the conformations obtained from the pseudorotation maps.

The pseudorotation maps reveal the effect of the substitution of the pyrrolidine ring on its conformation. The conformation of phosphonomethyl derivatives **7** and **9** with *trans* configuration on 3’ and 4’ carbon atoms tends to occupy mainly (~60%) the ^1^T_2_ or ^1^E conformation in an equilibrium with the less populated (~40%) _1_E conformer. This equilibrium is significantly shifted in the case of *cis* derivatives **8** and **10** towards the _2_T^3^ conformation (>80%) due to the steric hindrance of 3’ and 4’ substituents, where the nucleobase occupies predominantly the pseudo-equatorial position. There is an obvious contrast between minor conformations of **8** and **10**, which might be caused by hydrogen bonding between the 3’-OH group and the 4’-nucleobase substituent.

A similar effect in the conformer populations was observed for phosphonoformyl derivatives. While *trans* derivatives **11** and **13** exist as an about 1:1 equilibrium mixture of ^3^T_4_ and _3_T^4^ conformations, *cis* derivatives **12** and **14** prefer (>80%) the ^3^T_4_ conformation. The orientation of the phosphonoformyl moiety (rotamers A and B) has a negligible effect on the conformation of the pyrrolidine ring; therefore, only conformation maps for rotamer A are presented in Table 3. The effect of the alkylation or acylation of the pyrrolidine nitrogen is also fairly visible in the pseudorotation maps. Phosphonoformyl derivatives **11–14** keep the conformation of the pyrrolidine ring in narrow northern (*P* ~ 0°) and southern (P ~ 180°) regions, which arises from the plane arrangement of the C2’–N1’–C5’ fragment of the pyrrolidine ring (minor conformers of *cis* derivatives **12A** and **14A** do not follow this trend). In contrast to that, the conformation of the pyrrolidine ring in phosphonomethyl derivatives **7–10** is more flexible and rather occupies the eastern and western segments of the pseudorotation wheel, reacting sensitively to the hydrogen bonding between the 3’-OH group and the 4’-nucleobase substituent.

**Figure 13:**
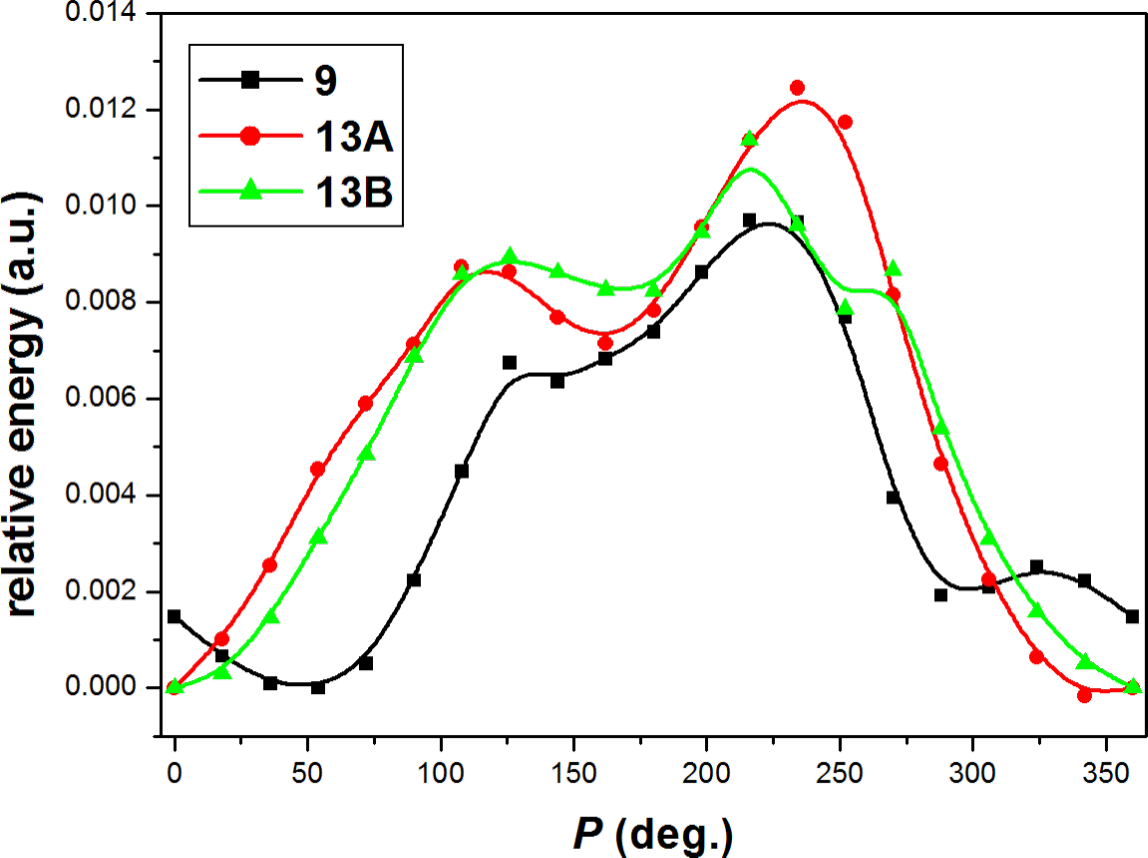
The energy profile of the five-membered pyrrolidine ring pseudorotation for adenine derivatives **9** and **13**.

In order to supplement our experimental observation, we calculated the energy profile of the five-membered pyrrolidine ring pseudorotation for adenine derivatives **9** and **13**. We generated 20 conformers covering the whole pseudorotation pathway in 18-degree steps with constant maximum puckering amplitude of 40 degrees. For each conformer, the endocyclic torsion angles Φ_0_ and Φ_3_ were kept fixed and the geometry of the molecule was optimized using the DFT B3LYP/6-31G* method in vacuo. Predominant conformations can be found as the energy minima by plotting the calculated energy against phase angle *P* (Figure 13). The curves in Figure 13 proved a two-state equilibrium used as a prerequisite in PSEUROT-based programs and known for furanose conformation in natural nucleosides and nucleotides.

**Figure 14:**
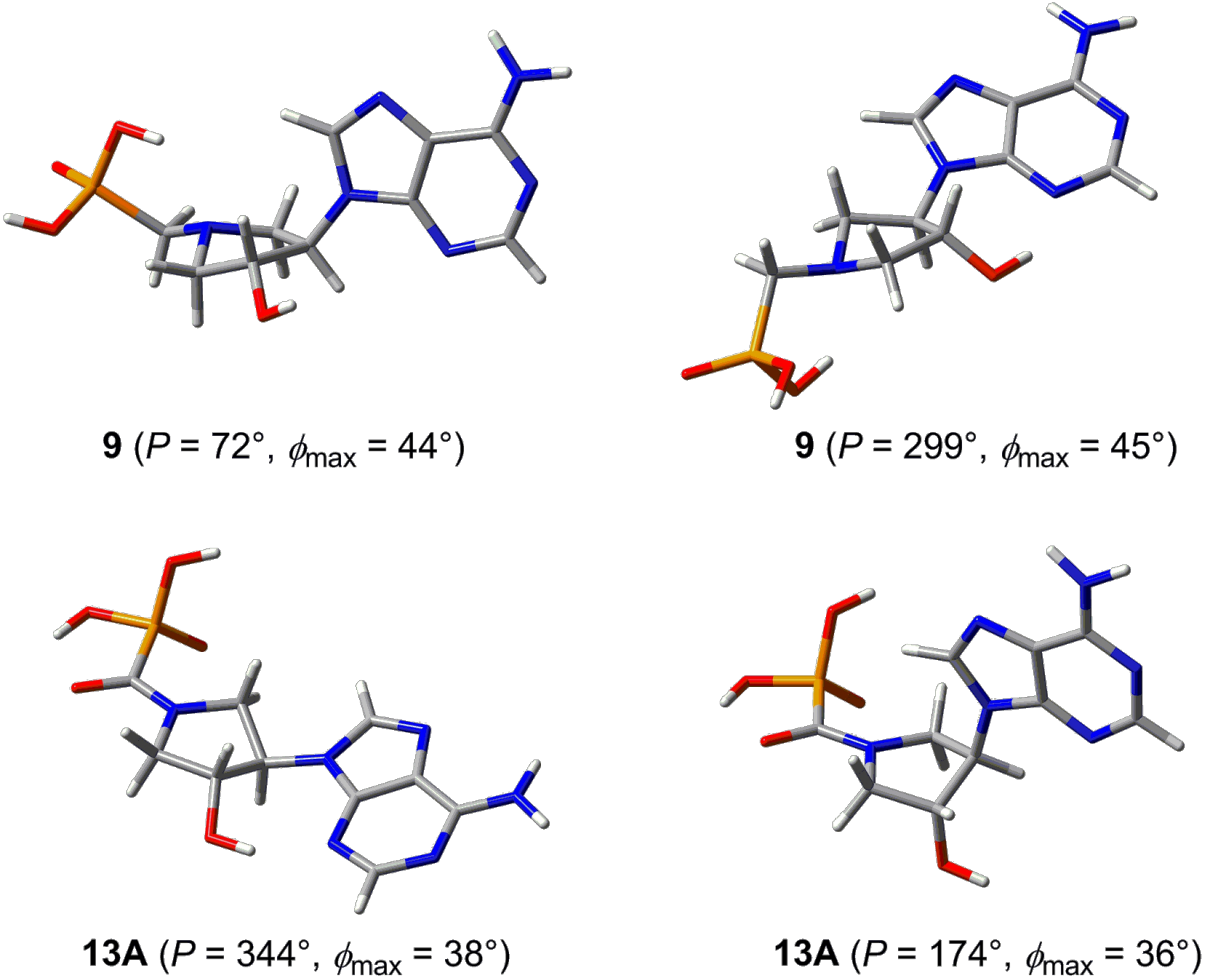
The most stable conformations of adenine derivatives **9** and **13A** calculated by the B3LYP/6-31++G* method in water (CPCM).

The two energy minima were then fully optimized by B3LYP/6-31++G* in water (the CPCM model). The optimized conformations were compared with those obtained by conformational analysis using ^3^*J*_HH_ and the results showed a good agreement of the molecular modeling and the experiment (cf. Table 3 and Figure 14).

## Conclusion

In summary, we presented an NMR and DFT conformational analysis of pyrrolidine nucleotide analogs bearing phosphonomethyl or phosphonoformyl substituents attached to the pyrrolidine nitrogen atom. The mode of the phosphonate moiety attachment to the pyrrolidine ring results in tuning the conformation of the five-membered pyrrolidine ring over the whole pseudorotation wheel. While phosphonoformyl derivatives **11–14** keep the conformation of the pyrrolidine ring in narrow northern (*P* ~ 0°) and southern (*P* ~ 180°) regions, which arises from the plane arrangement of the C2’–N1’–C5’ fragment of the pyrrolidine ring, the conformation of phosphonomethyl derivatives **7–10** is more flexible and rather occupies the eastern and western segment of the pseudorotation wheel. In general, this simple yet effective tuning of the pyrrolidine-ring conformation can be used for a number of other pyrrolidine derivatives.

## Experimental

### Chemistry

Phosphonoalkyl derivatives **7**–**10** were prepared according to the procedures described in [20] while thymine carbonylphosphonic acids **11** and **12** were prepared according to the procedures described in [4]. Unless stated otherwise, all solvents used were anhydrous. TLC was performed on TLC plates precoated with silica gel (silica gel/TLC-cards, UV 254, Merck). The compounds were detected using UV light (254 nm), by spraying with 1% ethanolic solution of ninhydrine to visualize amines, or by spraying with a 1% solution of 4-(4-nitrobenzyl)pyridine in ethanol, followed by heating and treatment with gaseous ammonia (for the detection of alkylating agents, such as dialkyl phosphonates; blue color). The course of the reactions and the purity of the prepared compounds were determined by LC–MS using a Waters AutoPurification System with a 2545 Quaternary Gradient Module and a 3100 Single Quadrupole Mass Detector using a Luna C18 column (100 × 4.6 mm, 3 µm, Phenomenex) at a flow rate of 1 mL/min. The following mobile phase was used, where A, B, and C represent 50 mM NH_4_HCO_3_, 50 mM NH_4_HCO_3_ in 50% aq CH_3_CN, and CH_3_CN, respectively: A→B over 10 min, B→C over 10 min and C for 5 min. Preparative RP HPLC was performed on an LC5000 Liquid Chromatograph (INGOS-PIKRON, CR) using a Luna C18 (2) column (250 × 21.2 mm, 5 µm) at a flow rate of 10 mL/min. A gradient elution of methanol in pH 7.5 0.1 M TEAB (A, 0.1 M TEAB; B, 0.1 M TEAB in 50% aq methanol; C, methanol) was used. All final compounds were lyophilized. Mass spectra were collected on an LTQ Orbitrap XL (Thermo Fisher Scientific) using ESI ionization. The pD titrations were performed on pH meter IQ150 Scientific Instruments equipped with a pH probe PH47-SS. NMR spectra were acquired in D_2_O on a Bruker AVANCE 600 (^1^H at 600.1 MHz and ^13^C at 150.9 MHz), Bruker AVANCE 500 (^1^H at 499.8 MHz, ^13^C at 125.7 MHz and ^31^P at 202.3 MHz) and/or Bruker AVANCE 400 (^1^H at 400.0 MHz, ^13^C at 100.6 MHz and ^31^P at 162.0 MHz) NMR spectrometers. Chemical shifts (in ppm, δ scale) were referenced to the 1,4-dioxane signal (3.75 ppm for ^1^H and 69.3 ppm for ^13^C) as an internal or external standard. ^31^P NMR spectra were referenced to H_3_PO_4_ (0 ppm) as an external standard. Coupling constants (*J*) are given in Hz. The complete assignment of ^1^H and ^13^C signals was performed by an analysis of the correlated homonuclear H,H-COSY, H,H-ROESY and heteronuclear H,C-HSQC and H,C-HMBC spectra.

#### Calculations

All calculations were carried out using the Gaussian 09 software package [21]. DFT calculations were performed using Becke3-LYP [22–23] with 6-31G* basis set for a fixed conformation geometry in the calculation of the energy profile (Figure 13) or 6-31++G* basis set for full geometry optimizations. The full geometry optimizations were carried out using conductor-like polarizable continuum model (CPCM) [24].

#### General method 1: Synthesis of phosphonoacyl derivatives (**13** and **14**)

General method 1 followed the similar procedure described previously [6]. The mixture of pyrrolidine nucleosides **15a,b** [15] (1 mmol) and diisopropyl phenylphosphonoformiate (1.2 mmol) in DMF (10 mL) was stirred at 80 °C for 1 h (the course of the reaction was followed by TLC using the solvent system H1 (EtOAc/acetone/EtOH/H_2_O 4:1:1:1) or LC–MS). The reaction mixture was concentrated in vacuo and the intermediate **18a,b** was obtained in a pure form by chromatography on silica gel using a linear gradient of H1 in ethyl acetate. The intermediate **18a,b** (1 mmol) was dissolved in acetonitrile (10 mL) and bromotrimethylsilane (5 mmol) was added under argon atmosphere. The reaction mixture was stirred overnight at room temperature under argon atmosphere. The reaction mixture was concentrated in vacuo, dissolved in 0.5 M aqueous TEAB (5 mL) and evaporated. The final product was obtained by preparative HPLC, converted to its sodium salt by passing through a column of Dowex 50 in a Na^+^ form (10 mL/mmol) and lyophilized from water.

#### (3*R,*4*R*)-4-(Adenin-9-yl)-3-hydroxypyrrolidin-1-*N*-ylcarbonylphosphonic acid (**13**)

The title compound was prepared according to general method 1 from pyrrolidine nucleoside **15a** (0.08 g, 0.36 mmol) in 56% overall yield (75.2 mg, 0.2 mmol) in the form of a white amorphous solid. HRMS–ESI: [M + H]^+^ calcd for C_10_H_13_O_5_N_6_NaP, 351.05736; found, 351.05728. The NMR spectra showed a 10:11 mixture of rotamers A:B. Rotamer A: ^1^H NMR (600.1 MHz, D_2_O, *T* = 25 °C, pD = 6.1) 3.54 (dddd, *J*_gem_ = 13.6, *J*_2",3'_ = 4.1, *J*_H,P_ = 1.9, *J*_2",5"_ = 0.9, 1H, H-2"), 3.83 (ddd, *J*_gem_ = 13.6, *J*_2',3'_ = 6.1, *J*_H,P_ = 1.9, 1H, H-2'), 4.55 (ddd, *J*_gem_ = 13.0, *J*_5',4'_ = 5.6, *J*_5',3'_ = 0.9, 1H, H-5'), 4.57 (ddd, *J*_gem_ = 13.0, *J*_5",4'_ = 6.3, *J*_5",2"_ = 0.9, 1H, H-5"), 4.73 (dddd, *J*_3',2'_ = 6.1, *J*_3',4'_ = 4.5, *J*_3',2"_ = 4.1, *J*_3',5'_ = 0.9, 1H, H-3'), 5.01 (ddd, *J*_4',5"_ = 6.3, *J*_4',5'_ = 5.6, *J*_4',3'_ = 4.5, 1H, H-4'), 8.134 (s, 1H, H-8), 8.151 (s, 1H, H-2); ^13^C NMR (150.9 MHz, D_2_O, *T* = 25 °C, pD = 6.1) 51.65 (C-5'), 53.11 (d, *J*_C,P_ = 4.6, C-2'), 62.87 (C-4'), 73.75 (C-3'), 121.29 (C-5), 142.56 (C-8), 151.58 (C-4), 154.92 (C-2), 157.98 (C-6), 179.15 (d, *J*_C,P_ = 197.5, P-CO); ^31^P{1H} NMR (202.3 MHz, D_2_O, *T* = 25 °C, pD = 6.1) −2.25. Rotamer B: ^1^H NMR (600.1 MHz, D_2_O, *T* = 25 °C, pD = 6.1) 3.95 (dd, *J*_gem_ = 12.6, *J*_2",3'_ = 5.2, 1H, H-2"), 4.01 (ddd, *J*_gem_ = 13.6, *J*_5',4'_ = 5.5, *J*_H,P_ = 1.9, 1H, H-5'), 4.19 (ddd, *J*_gem_ = 13.6, *J*_5",4'_ = 7.8, *J*_H,P_ = 2.0, 1H, H-5"), 4.31 (ddd, *J*_gem_ = 12.6, *J*_2',3'_ = 5.8, *J*_2',5'_ = 0.8, 1H, H-2'), 4.76 (ddd, *J*_3',2'_ = 5.8, *J*_3',4'_ = 5.3, *J*_3',2"_ = 5.2, 1H, H-3'), 4.99 (ddd, *J*_4',5"_ = 7.8, *J*_4',5'_ = 5.5, *J*_4',3'_ = 5.3, 1H, H-4'), 8.130 (s, 1H, H-8), 8.149 (s, 1H, H-2); ^13^C NMR (150.9 MHz, D_2_O, *T* = 25 °C, pD = 6.1) 50.23 (d, *J*_C,P_ = 4.9, C-5'), 54.64 (C-2'), 60.88 (C-4'), 75.44 (C-3'), 121.29 (C-5), 142.73 (C-8), 151.62 (C-4), 154.95 (C-2), 158.00 (C-6), 179.24 (d, *J*_C,P_ = 197.0, P-CO); ^31^P{1H} NMR (202.3 MHz, D_2_O, *T* = 25 °C, pD = 6.1) −2.23.

#### (3*S,*4*R*)-4-(Adenin-9-yl)-3-hydroxypyrrolidin-1-*N*-ylcarbonylphosphonic acid (**14**)

The title compound was prepared according to general method 1 from pyrrolidine nucleoside **15b** (0.19 g, 0.86 mmol) in 28% overall yield (91 mg, 0.25 mmol) in the form of a white amorphous solid. HRMS–ESI: [M + H]^+^ calcd for C_10_H_13_O_5_N_6_NaP, 351.05736; found, 351.05773. The NMR spectra showed a 10:11 mixture of rotamers A:B. Rotamer A: ^1^H NMR (600.1 MHz, D_2_O, *T* = 25 °C, pD = 5.9) 3.65 (dt, *J*_gem_ = 13.7, *J*_2',3'_ = *J*_H,P_ = 2.1, 1H, H-2'), 3.90 (ddd, *J*_gem_ = 13.7, *J*_2",3'_ = 5.1, *J*_H,P_ = 1.9, 1H, H-2"), 4.41 (dd, *J*_gem_ = 11.6, *J*_5',4'_ = 9.2, 1H, H-5'), 4.66 (dd, *J*_gem_ = 11.6, *J*_5",4'_ = 7.6, 1H, H-5"), 4.693 (ddd, *J*_3',2"_ = 5.1, *J*_3',4'_ = 4.2, *J*_3',2'_ = 2.1, 1H, H-3'), 5.19 (ddd, *J*_4',5'_ = 9.2, *J*_4',5"_ = 7.6, *J*_4',3'_ = 4.2, 1H, H-4'), 8.158 (s, 1H, H-2), 8.262 (s, 1H, H-8); ^13^C NMR (150.9 MHz, D_2_O, *T* = 25 °C, pD = 5.9) 50.18 (C-5'), 54.30 (d, *J*_C,P_ = 4.5, C-2'), 58.95 (C-4'), 70.35 (C-3'), 120.80 (C-5), 143.65 (C-8), 151.93 (C-4), 154.89 (C-2), 157.95 (C-6), 178.88 (d, *J*_C,P_ = 198.3, P-CO); ^31^P{^1^H} NMR (202.3 MHz, D_2_O, *T* = 25 °C, pD = 5.9) −2.21. Rotamer B: ^1^H NMR (600.1 MHz, D_2_O, *T* = 25 °C, pD = 5.9) 4.04 (ddd, *J*_gem_ = 12.8, *J*_5',4'_ = 8.3, *J*_H,P_ = 1.9, 1H, H-5'), 4.14 (dd, *J*_gem_ = 13.0, *J*_2',3'_ = 3.5, 1H, H-2'), 4.18 (ddd, *J*_gem_ = 12.8, *J*_5",4'_ = 8.2, *J*_H,P_ = 1.8, 1H, H-5"), 4.20 (dd, *J*_gem_ = 12.6, *J*_2",3'_ = 4.6, 1H, H-2"), 4.698 (ddd, *J*_3',2"_ = 4.6, *J*_3',4'_ = 4.3, *J*_3',2'_ = 3.5, 1H, H-3'), 5.21 (ddd, *J*_4',5'_ = 8.3, *J*_4',5"_ = 8.2, *J*_4',3'_ = 4.3, 1H, H-4'), 8.163 (s, 1H, H-2), 8.261 (s, 1H, H-8); ^13^C NMR (150.9 MHz, D_2_O, *T* = 25 °C, pD = 5.9) 49.06 (d, *J*_C,P_ = 5.2, C-5'), 55.63 (C-2'), 57.39 (C-4'), 72.34 (C-3'), 120.82 (C-5), 143.66 (C-8), 152.04 (C-4), 154.90 (C-2), 157.95 (C-6), 178.91 (d, *J*_C,P_ = 197.9, P-CO); ^31^P{^1^H} NMR (202.3 MHz, D_2_O, *T* = 25 °C, pD = 5.9) −2.30.

## Supporting Information

The pD titration curves of **9** and **10**, the variable temperature NMR of **14**, the pyrrolidine-ring parameterization for **9**, **10**, **13** and **14**, the NMR signal assignments and copies of the ^1^H, ^13^C and ^31^P NMR spectra are given.

File 1 Additional experimental data.
