# Supplementary material for: Pyrrolidine nucleotide analogs with a tunable conformation
Source: Beilstein J Org Chem. 2014 Aug 22;10:1967–80. doi: 10.3762/bjoc.10.205 (PMC4168946; doi:10.3762/bjoc.10.205)
Supplement: File 1 — Additional experimental data. [file Beilstein_J_Org_Chem-10-1967-s001.pdf]

Supporting Information

for

Pyrrolidine nucleotide analogs with a tunable conformation

Lenka Poštová Slavětínská, Dominik Rejman and Radek Pohl*

Address: Institute of Organic Chemistry and Biochemistry, Academy of Sciences of the Czech Republic, Flemingovo nám. 2, 166 10 Prague 6, Czech Republic

Email: Radek Pohl - pohl@uochb.cas.cz

* Corresponding author

Additional experimental data

Table of contents:

The pD titration curves for compounds **9** and **10** S2

Variable temperature ^{31}P NMR of **14** S3

The parametrization of the pyrrolidine ring for compounds **9**, **10**, **13A**, **13B**, **14A** and **14B** S4

NMR signals assignment and copies of ^{1}H, ^{13}C and ^{31}P NMR spectra S10

The pD titration curves for compounds 9 and 10

Compound 9

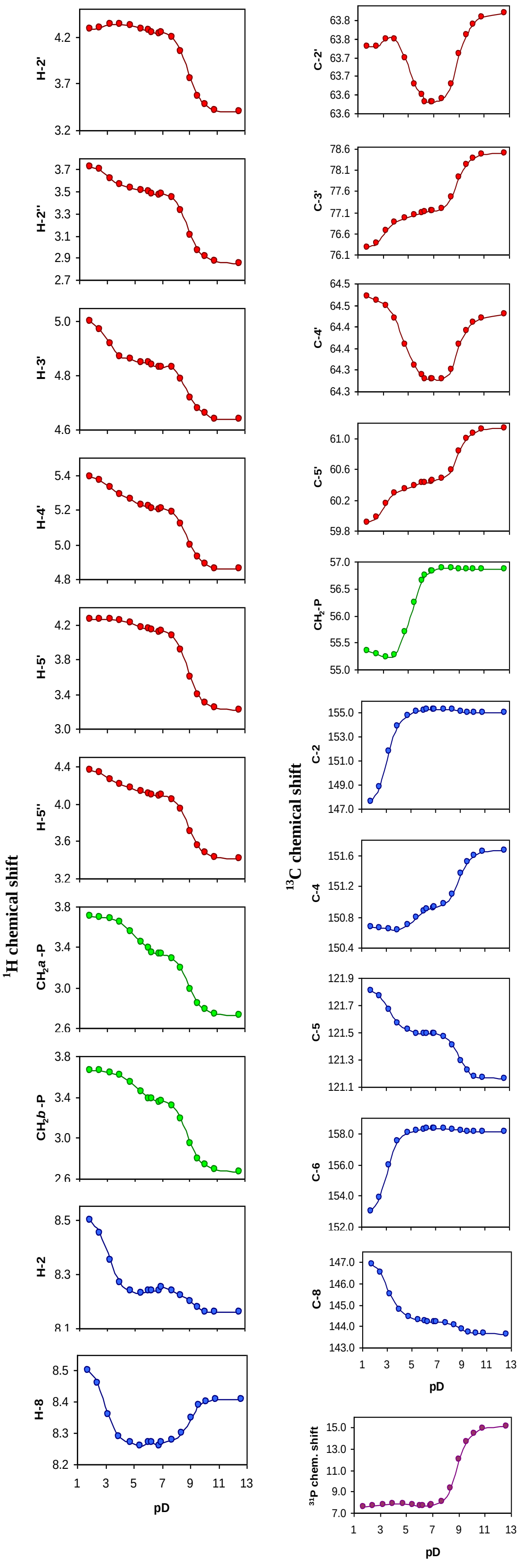

Compound 10

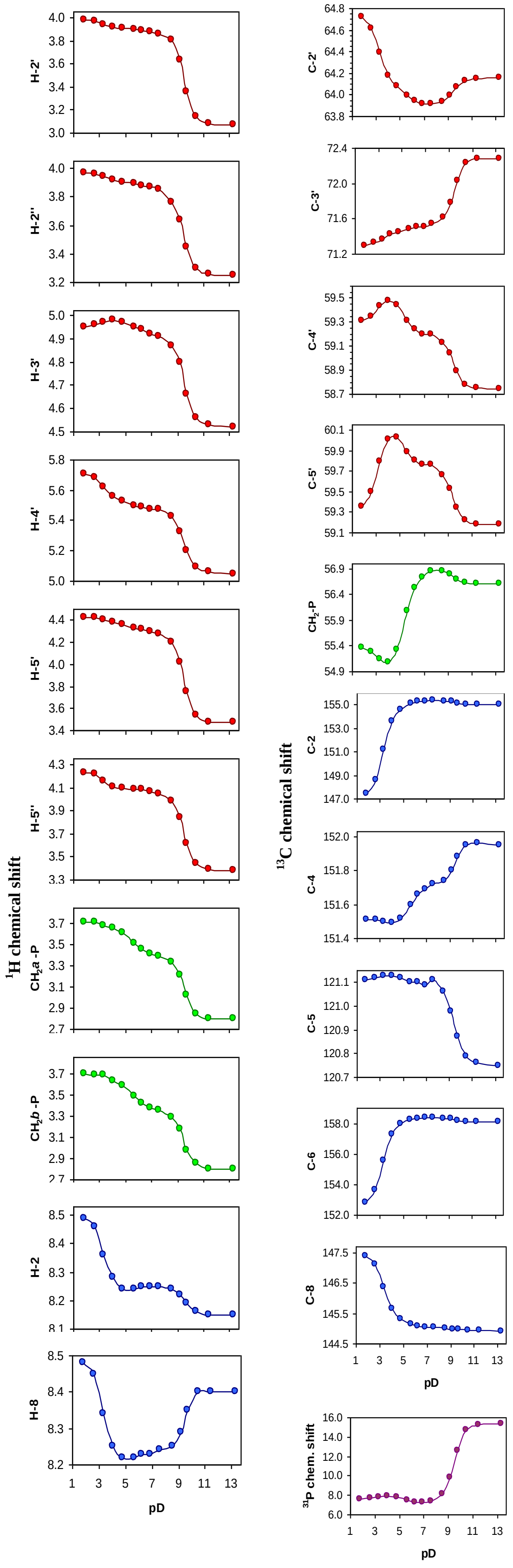

Variable temperature ^{31}P NMR of 14

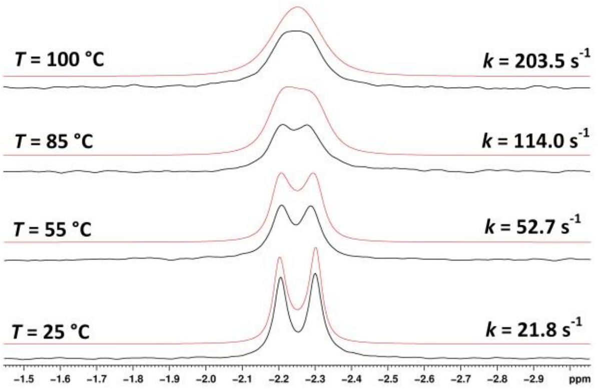

Figure S1: ^{31}P NMR spectra (202.3 MHz) of **14** measured (black curve) and simulated (red curve) at various temperatures.

Table S1: Rate constant values obtained by lineshape analysis of the spectra measured at various temperatures for **14**.

T (°C)	*T* (K)	1/*T* (K^{-1})	*k* (s^{-1})	ln(*k*/*T*)
25	298.15	0.003354	21.8	-2.61569
35	308.15	0.003245	30.1	-2.32606
45	318.15	0.003143	42.2	-2.02010
55	328.15	0.003047	52.7	-1.82886
65	338.15	0.002957	69.7	-1.57929
75	348.15	0.002872	98.5	-1.26258
85	358.15	0.002792	114.0	-1.14475
95	368.15	0.002716	164.5	-0.80558
100	373.15	0.002680	203.5	-0.60631

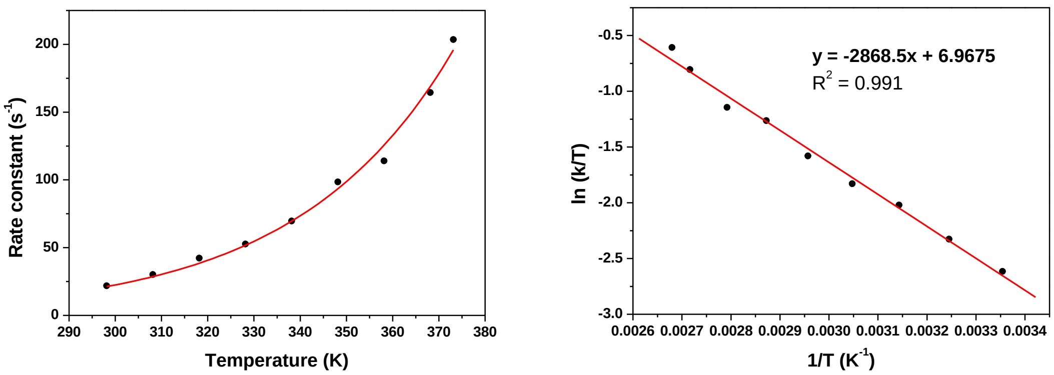

Figure S2: The plot of *T* vs. *k* and 1/*T* vs. ln(*k*/*T*) for compound **14**.

The parametrization of the pyrrolidine ring

The relation between exocyclic ϕ_{exo} and endocyclic ϕ_{endo} dihedral angles ($\phi_{exo} = A\phi_{endo} + B$) extracted from DFT B3LYP/6-31G* optimized geometries of 20 conformers.

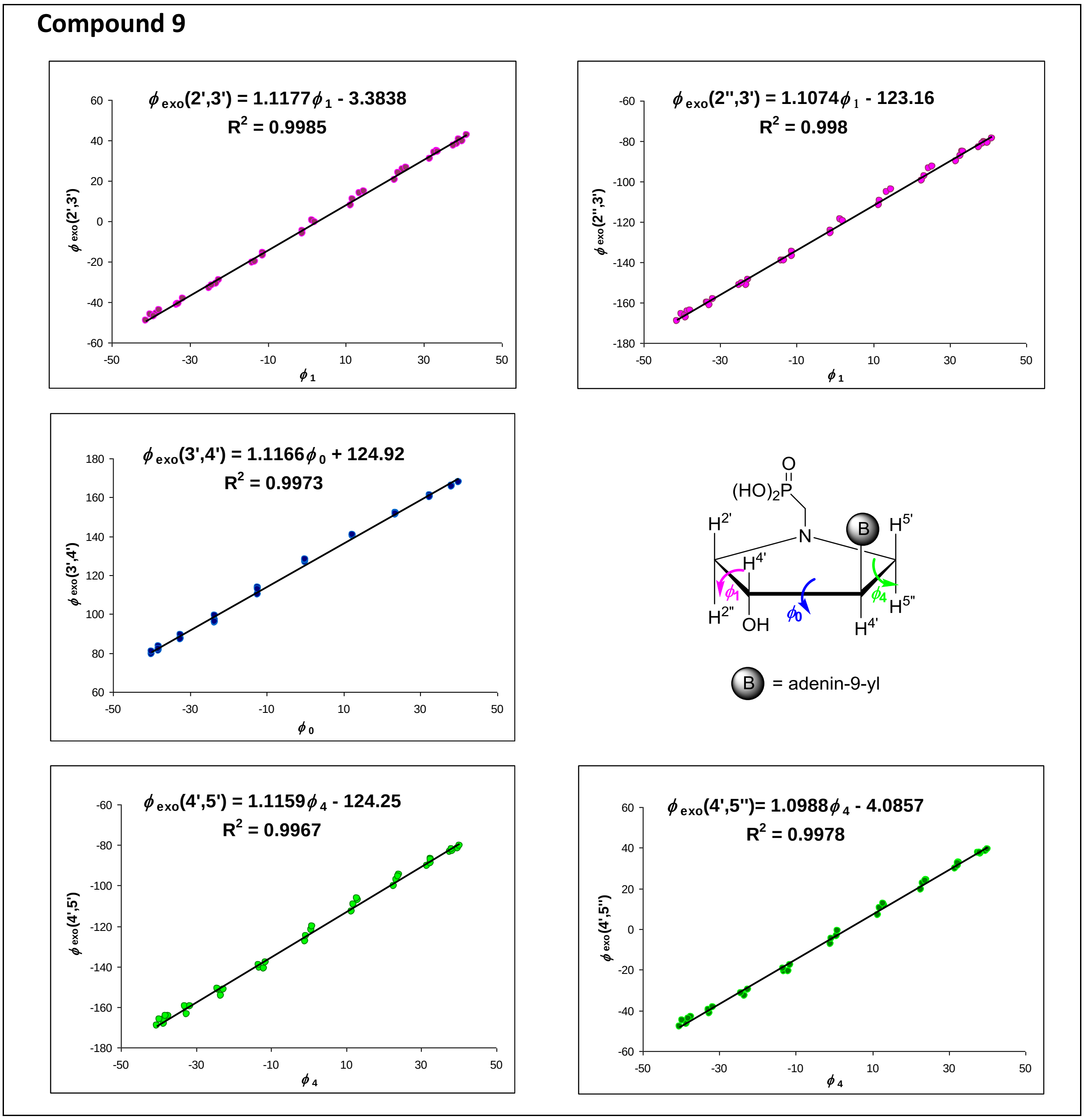

Figure S3: The ring parametrization for compound **9**.

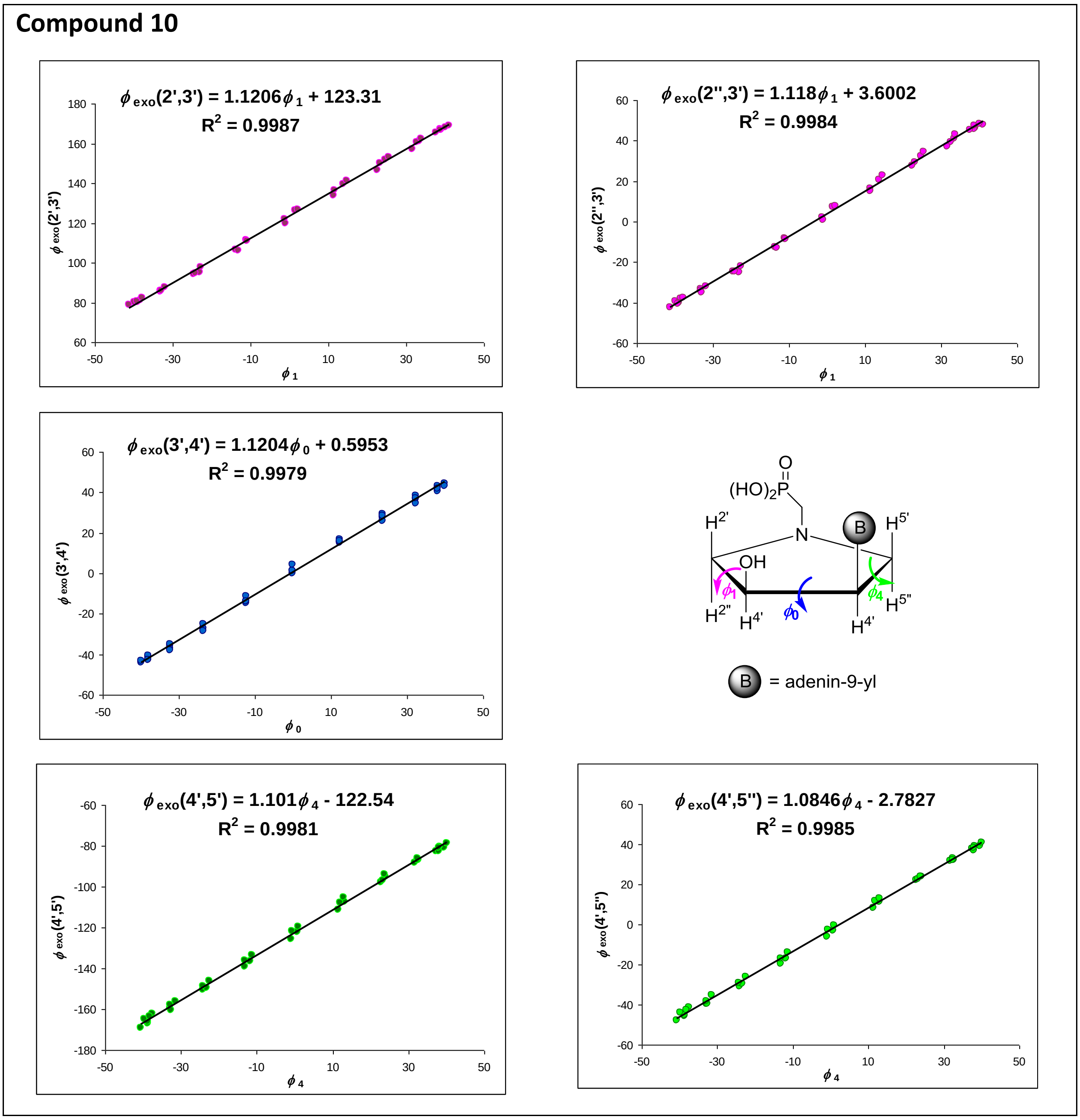

Figure S4: The ring parametrization for compound **10**.

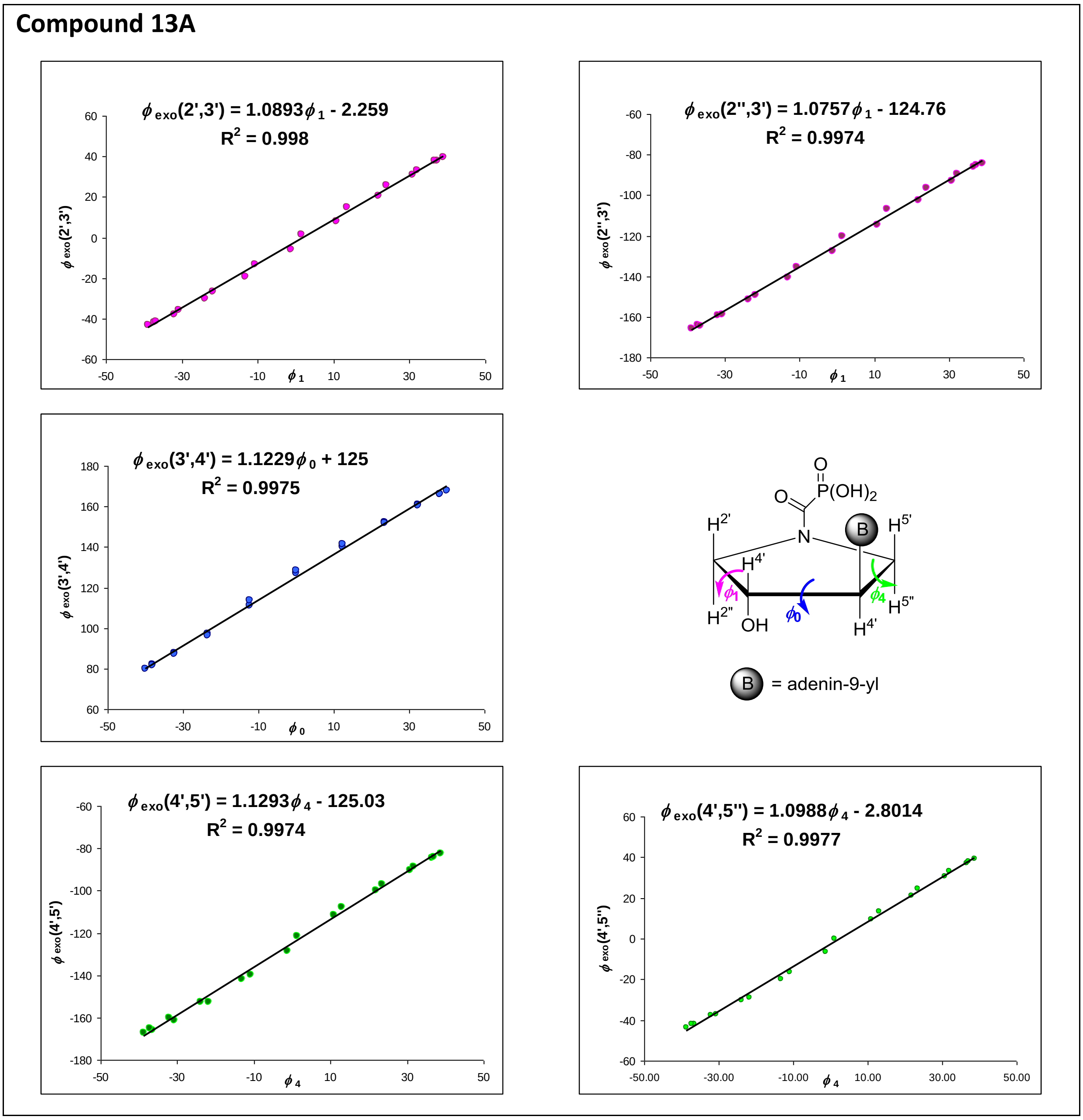

Figure S5: The ring parametrization for compound **13A**.

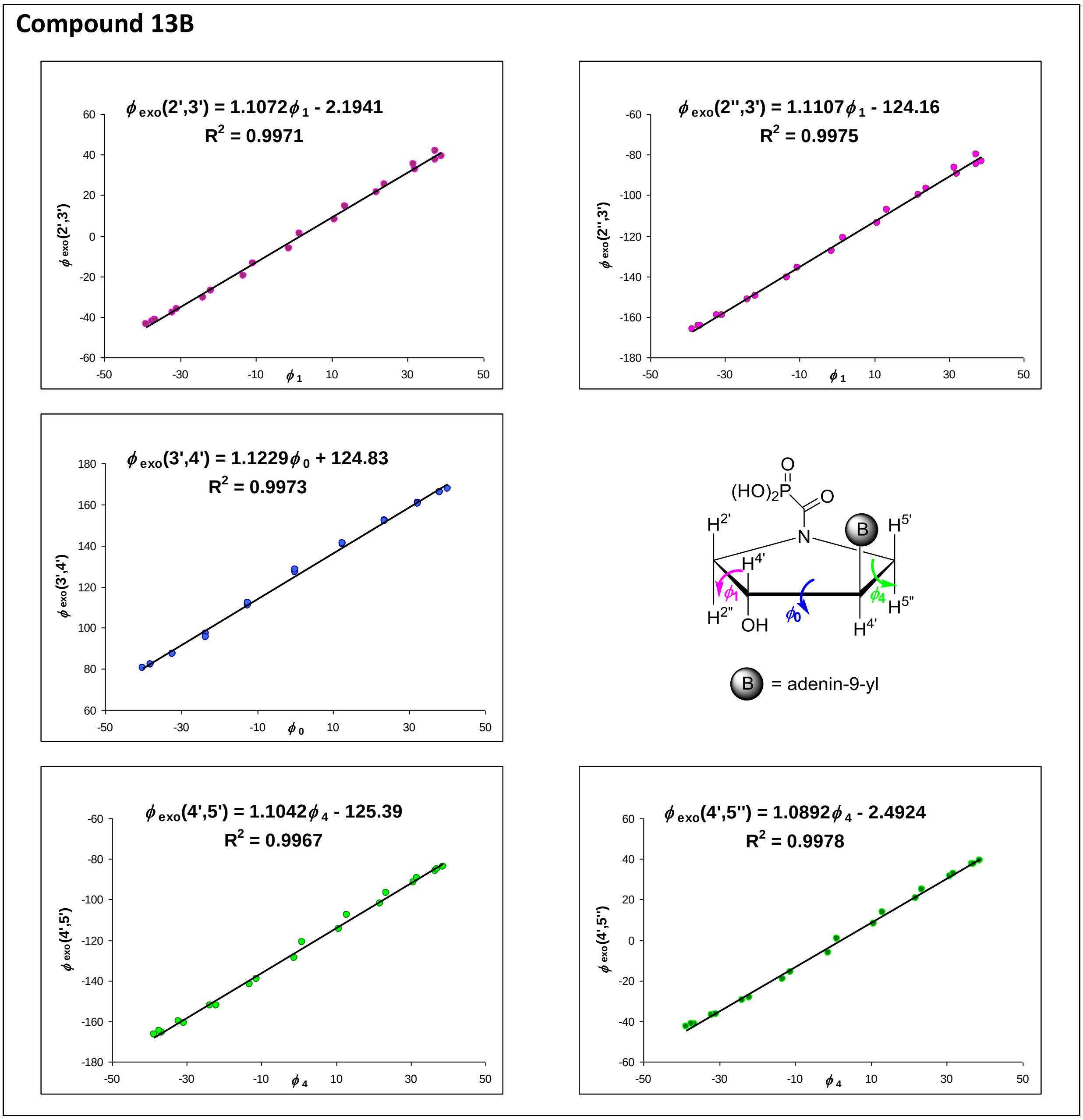

Figure S6: The ring parametrization for compound **13B**.

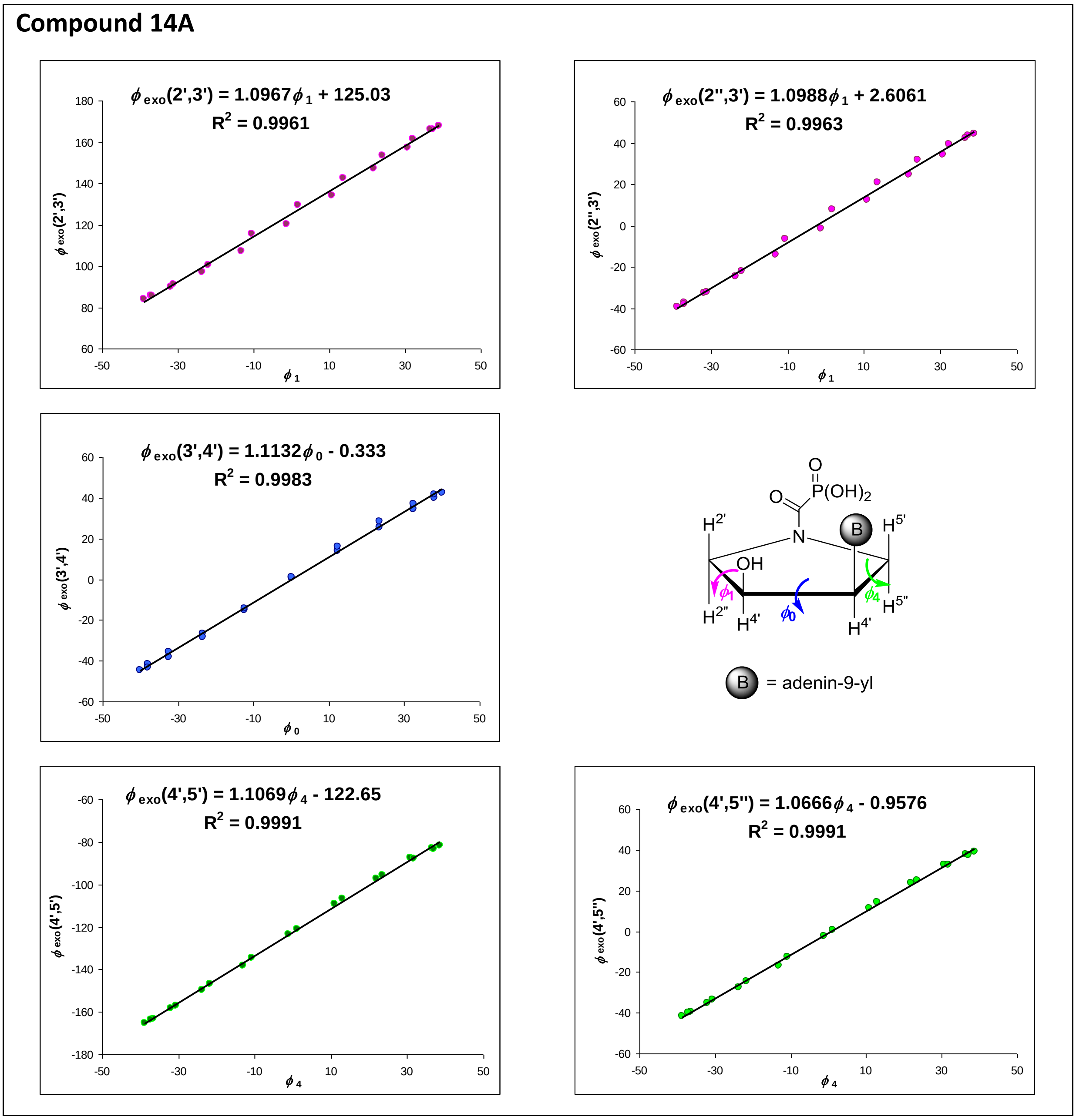

Figure S7: The ring parametrization for compound **14A**.

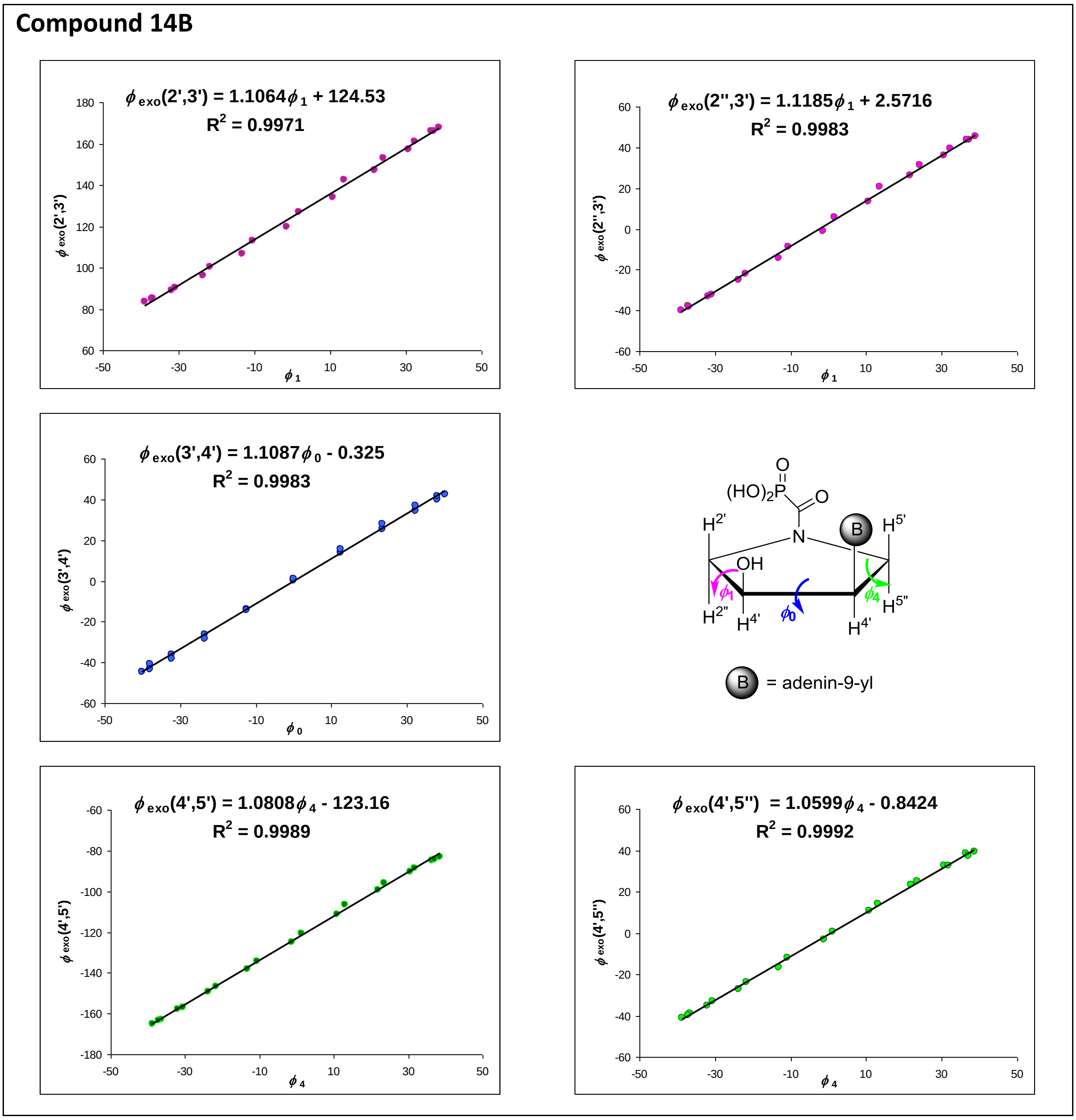

Figure S8: The ring parametrization for compound **14B**.

NMR signals assignment and copies of ^{1}H, ^{13}C and ^{31}P NMR spectra

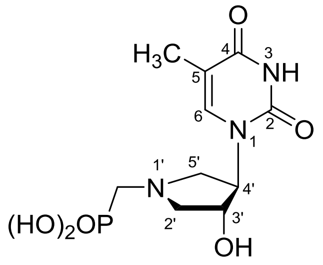

7

^{1}H NMR (499.8 MHz, D_2O, T = 25 °C, pD = 6.4): 1.86 (d, 3H, ^{4}J = 1.2, CH_3); 3.18 (dd, 1H, J_{gem} = 14.2, $J_{H,P}$ = 11.3, $CH_a\mathbf{H_b}P$); 3.24 (dd, 1H, J_{gem} = 14.2, $J_{H,P}$ = 11.7, $CH_a\mathbf{H_b}P$); 3.39 (dd, 1H, J_{gem} = 12.0, $J_{2'',3'}$ = 6.4, H-2''); 3.87 (dd, 1H, J_{gem} = 12.8, $J_{5',4'}$ = 5.6, H-5'); 3.93 (dd, 1H, J_{gem} = 12.8, $J_{5'',4'}$ = 9.1, H-5''); 4.04 (dd, 1H, J_{gem} = 12.0, $J_{2',3'}$ = 6.9, H-2'); 4.62 (ddd, 1H, $J_{4',5''}$ = 9.1, $J_{4',5'}$ = 5.6, $J_{4',3'}$ = 4.1, H-4'); 4.83 (ddd, 1H, $J_{3',2'}$ = 6.9, $J_{3',2''}$ = 6.4, $J_{3',4'}$ = 4.1, H-3'); 7.49 (q, 1H, ^{4}J = 1.2, H-6).

^{13}C NMR (125.7 MHz, D_2O, T = 25 °C, pD = 6.4): 14.04 (CH_3); 56.23 (d, $J_{C,P}$ = 128.1, CH_2P); 59.38 (d, $J_{C,P}$ = 4.6, C-5'); 63.72 (d, $J_{C,P}$ = 5.1, C-2'); 69.45 (C-4'); 75.05 (C-3'); 113.95 (C-5); 145.10 (C-6); 154.84 (C-2); 169.46 (C-4);.

^{31}P{1H} NMR (202.3 MHz, D_2O, T = 25 °C, pD = 6.4): 7.16.

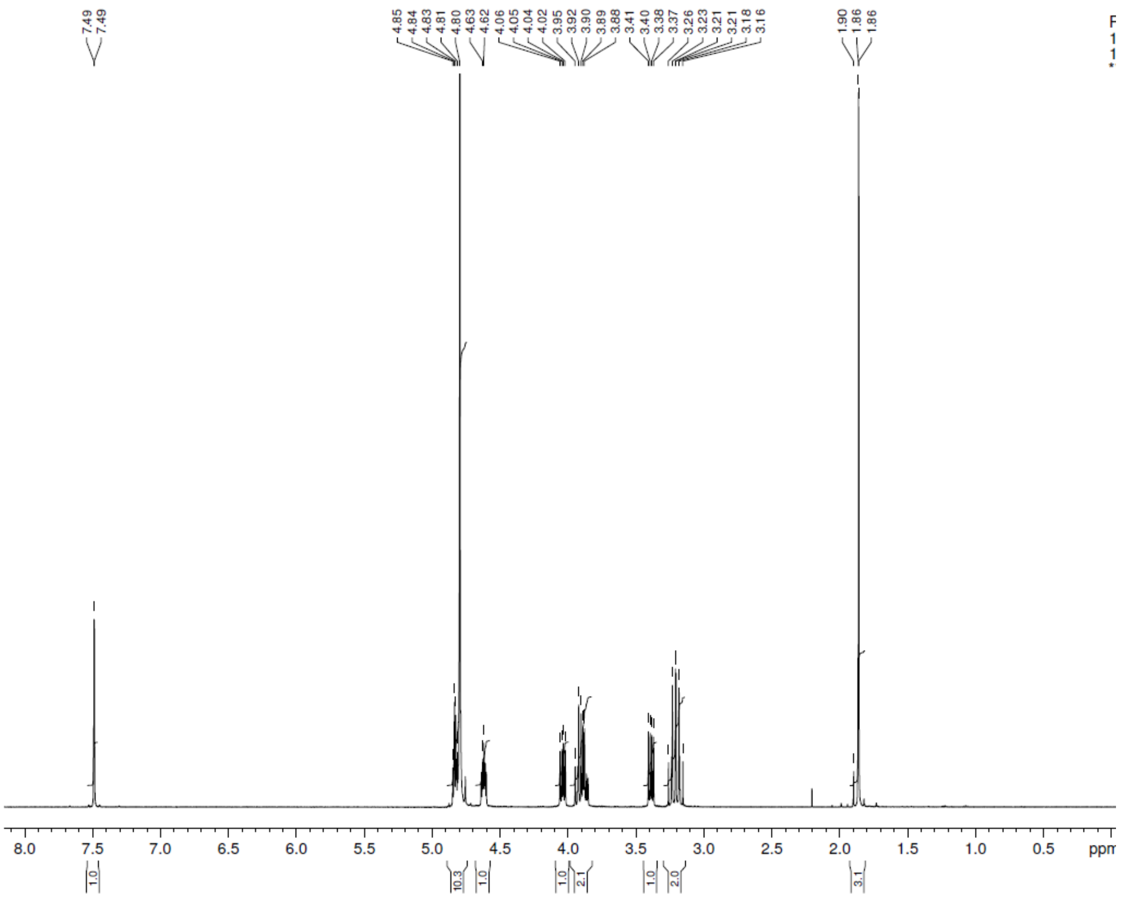

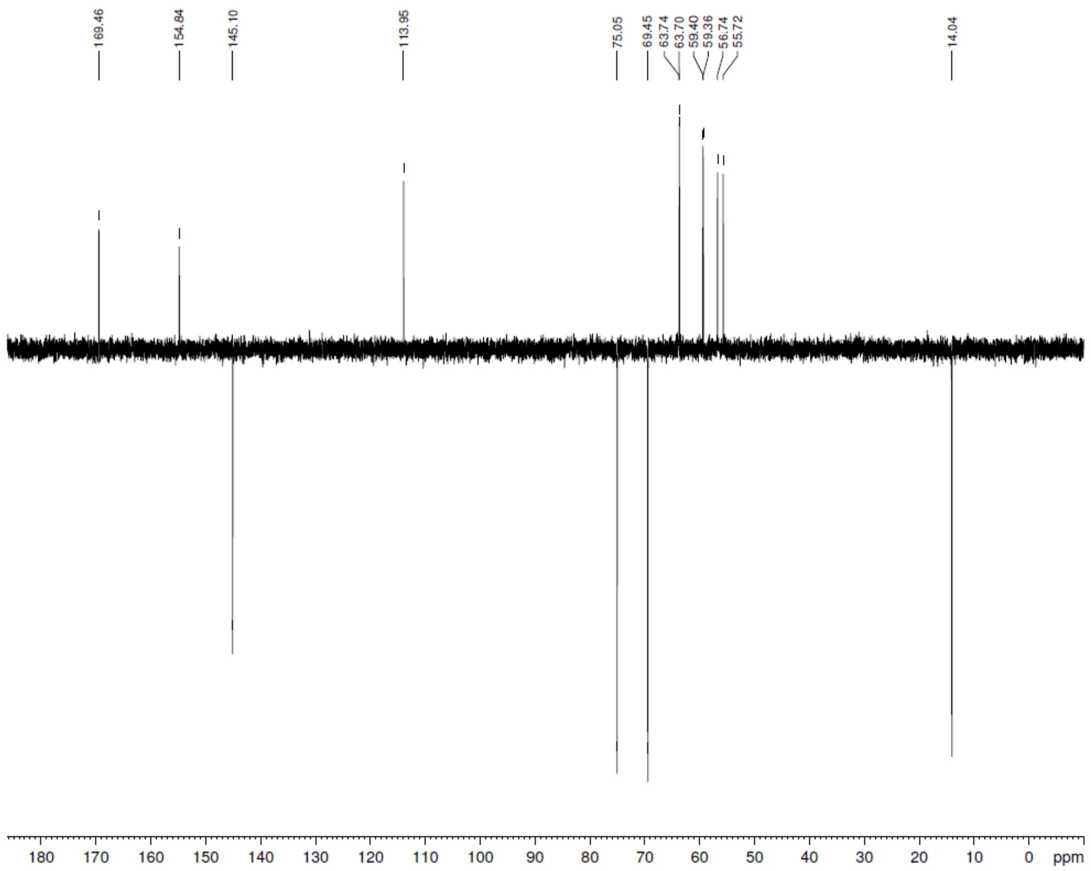
169.46
154.84
145.10
113.95
75.05
69.45
63.74
63.70
59.40
59.36
56.74
55.72
14.04
180
170
160
150
140
130
120
110
100
90
80
70
60
50
40
30
20
10
0
ppm

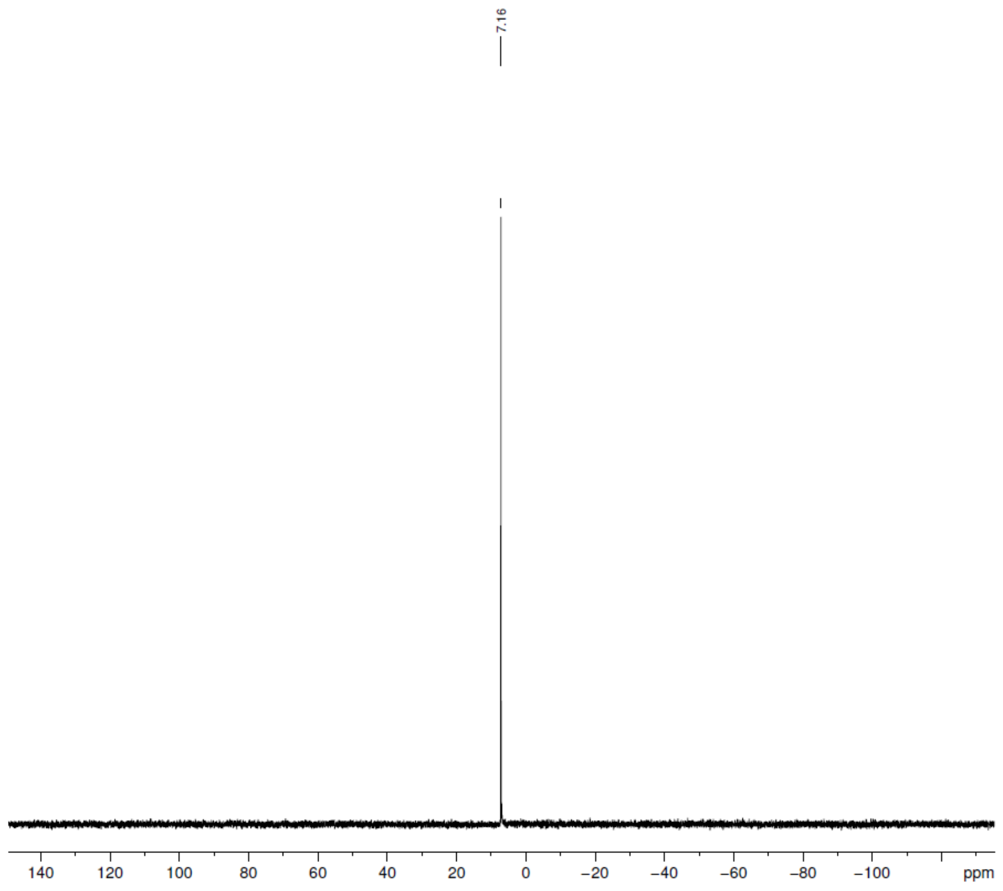
7.16
140
120
100
80
60
40
20
0
−20
−40
−60
−80
−100
ppm

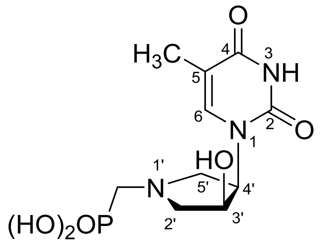

8

^{1}H NMR (499.8 MHz, D_2O, T = 25 °C, pD = 5.7): 1.88 (d, 3H, ^4J = 1.2, CH_3); 3.28, 3.31 (2 × dd, 2 × 1H, J_{gem} = 14.4, $J_{H,P}$ = 11.7, CH_2P); 3.68 (dd, 1H, J_{gem} = 12.7, $J_{2'',3'}$ = 5.2, H-2"); 3.78 (dd, 1H, J_{gem} = 12.7, $J_{2',3'}$ = 2.0, H-2'); 3.80 (dd, 1H, J_{gem} = 12.8, $J_{5'',4'}$ = 9.4, H-5"); 4.05 (dd, 1H, J_{gem} = 12.8, $J_{5',4'}$ = 6.9, H-5'); 4.71 (ddd, 1H, $J_{3',4'}$ = 6.0, $J_{3',2''}$ = 5.2, $J_{3',2'}$ = 2.0, H-3'); 5.19 (ddd, 1H, $J_{4',5''}$ = 9.4, $J_{4',5'}$ = 6.9, $J_{4',3'}$ = 6.0, H-4'); 7.58 (q, 1H, ^4J = 1.2, H-6).

^{13}C NMR (125.7 MHz, D_2O, T = 25 °C, pD = 5.7): 14.15 (CH_3); 56.21 (d, $J_{C,P}$ = 130.0, CH_2P); 58.13 (d, $J_{C,P}$ = 4.9, C-5'); 60.55 (C-4'); 64.95 (d, $J_{C,P}$ = 5.0, C-2'); 70.95 (C-3'); 113.28 (C-5); 144.39 (C-6); 155.36 (C-2); 169.23 (C-4);.

^{31}P{1H} NMR (202.3 MHz, D_2O, T = 25 °C, pD = 5.7): 7.29.

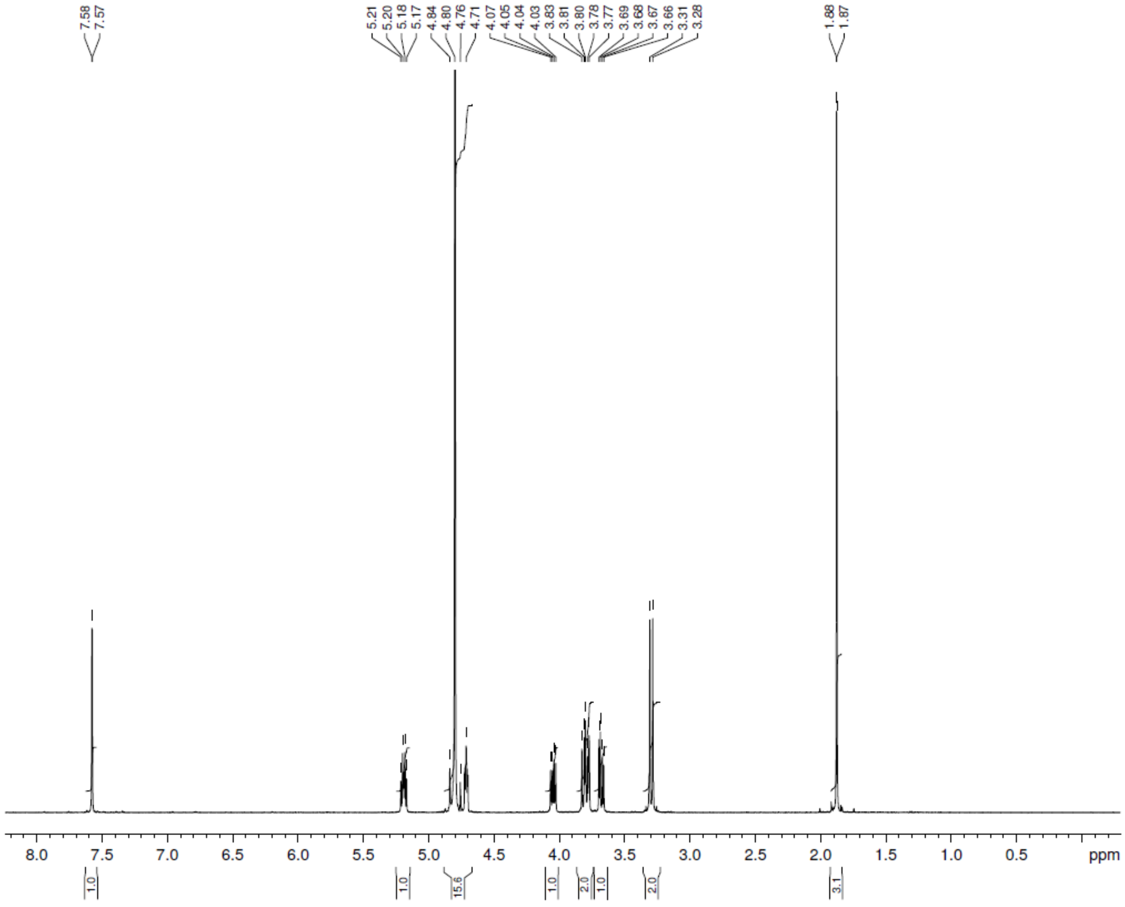

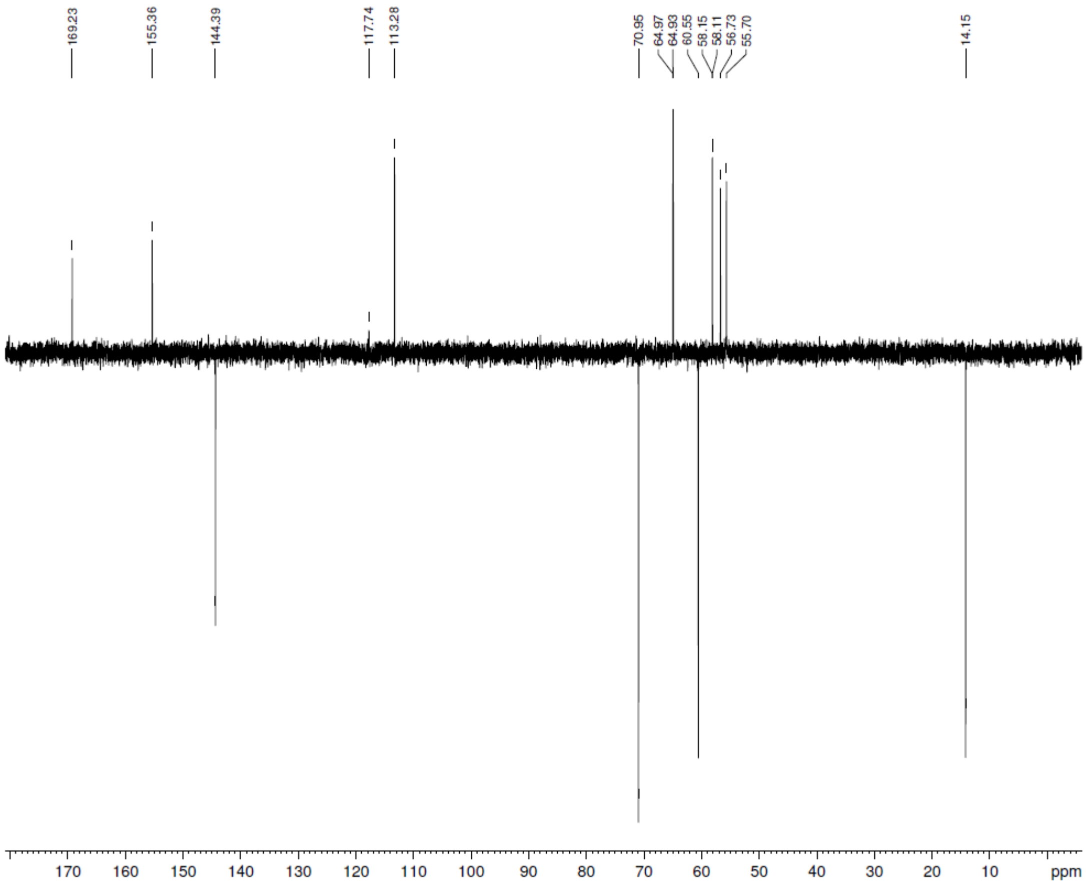
169.23
155.36
144.39
117.74
113.28
70.95
64.97
64.93
60.55
58.15
58.11
56.73
55.70
14.15
170 160 150 140 130 120 110 100 90 80 70 60 50 40 30 20 10 ppm

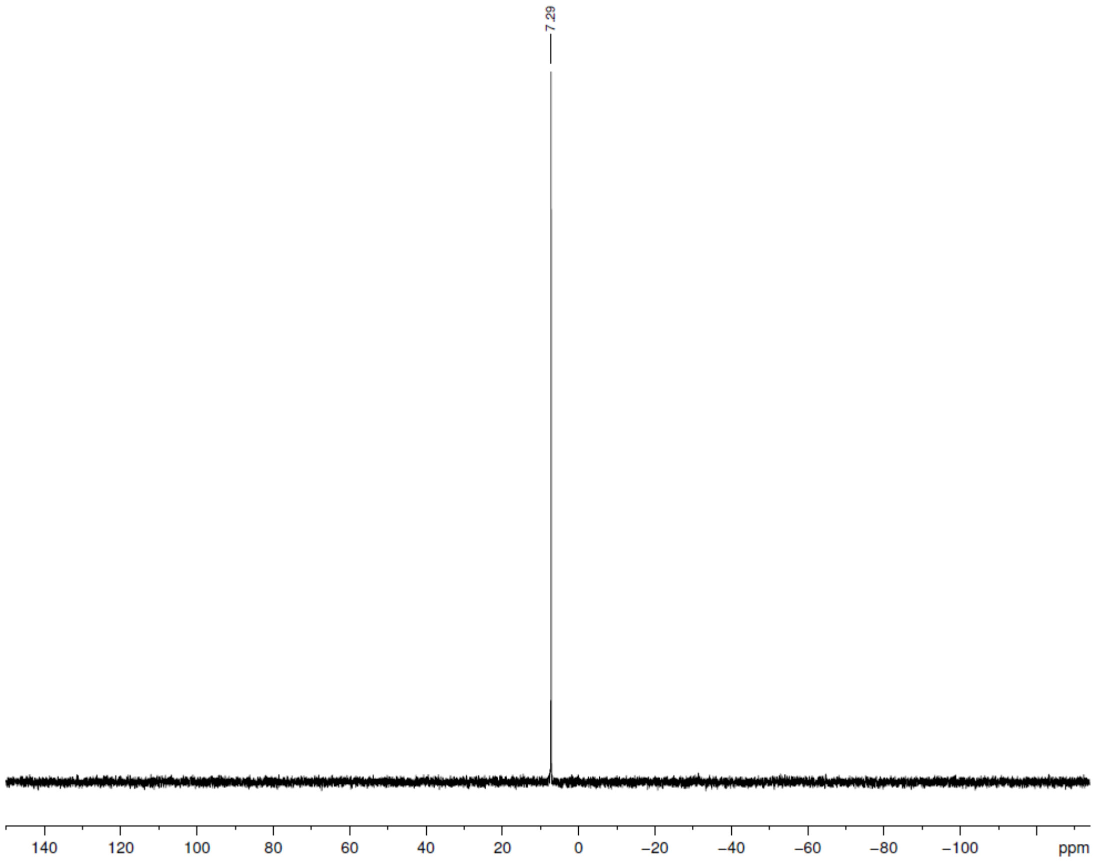
7.29
140 120 100 80 60 40 20 0 −20 −40 −60 −80 −100 ppm

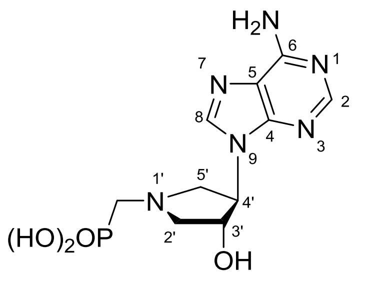

9

^{1}H NMR (499.8 MHz, D_2O, T = 25 °C, pD = 6.9): 3.33 (dd, 1H, J_{gem} = 14.4, $J_{H,P}$ = 11.3, CH$_a$**H$_b$**P); 3.36 (dd, 1H, J_{gem} = 14.4, $J_{H,P}$ = 11.4, C**H$_a$**H$_b$P); 3.48 (dd, 1H, J_{gem} = 12.6, $J_{2'',3'}$ = 5.1, H-2"); 4.09 (dd, 1H, J_{gem} = 13.0, $J_{5'',4'}$ = 7.3, H-5''); 4.13 (dd, 1H, J_{gem} = 13.0, $J_{5',4'}$ = 4.4, H-5'); 4.25 (dd, 1H, J_{gem} = 12.6, $J_{2',3'}$ = 6.5, H-2'); 4.78 (overlap with HDO signal, H-3'); 5.21 (ddd, 1H, $J_{4',5''}$ = 7.3, $J_{4',5'}$ = 4.4, $J_{4',3'}$ = 3.0, H-4'); 8.22 (s, 1H, H-8); 8.25 (s, 1H, H-2).

^{13}C NMR (125.7 MHz, D_2O, T = 25 °C, pD = 6.9): 56.83 (d, $J_{C,P}$ = 128.5, CH_2P); 60.45 (d, $J_{C,P}$ = 6.0, C-5'); 63.61 (d, $J_{C,P}$ = 4.3, C-2'); 64.28 (C-4'); 77.14 (C-3'); 121.49 (C-5); 144.20 (C-8); 150.93 (C-4); 155.31 (C-2); 158.31 (C-6).

^{31}P{1H} NMR (202.3 MHz, D_2O, T = 25 °C, pD = 6.9): 7.74.

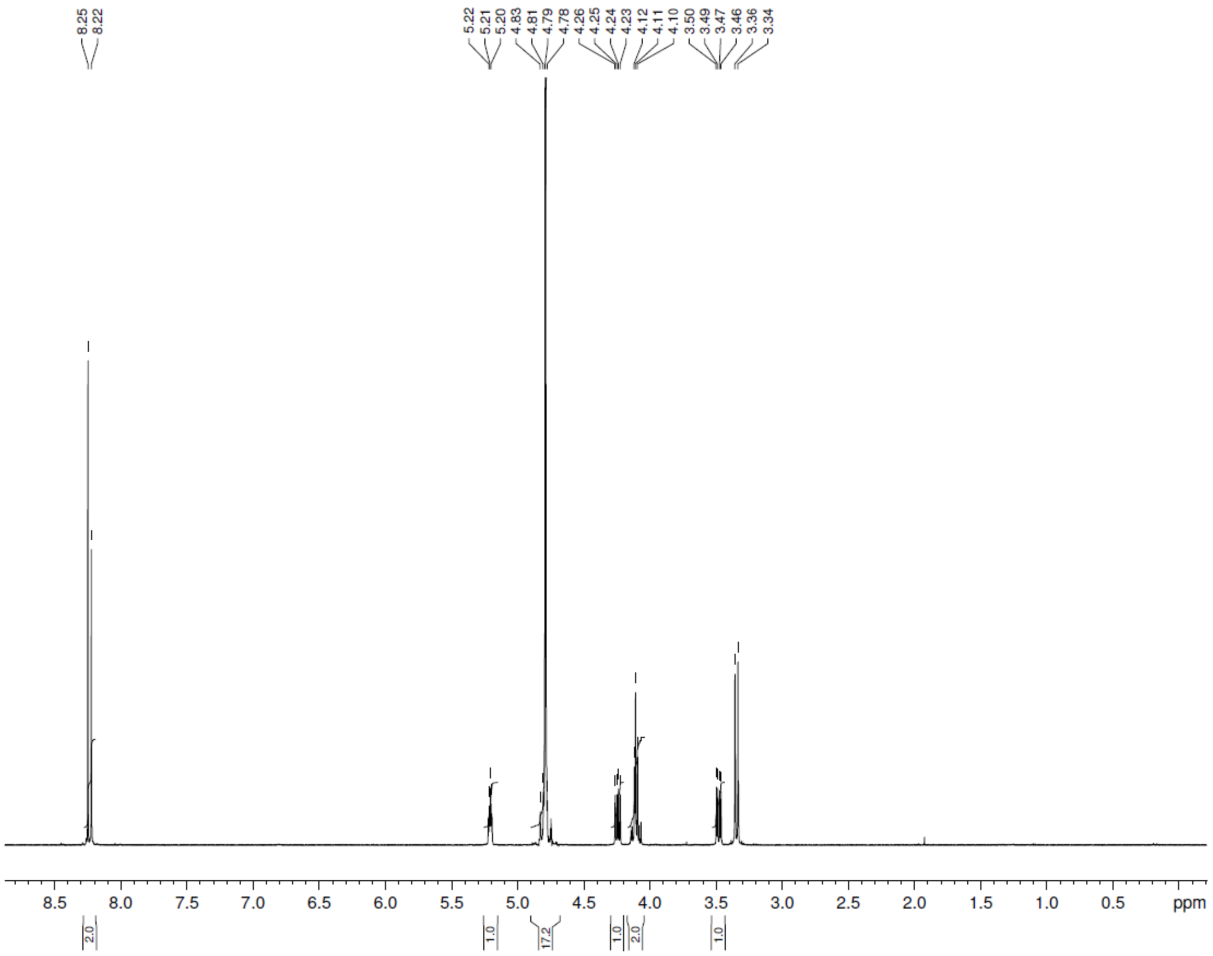

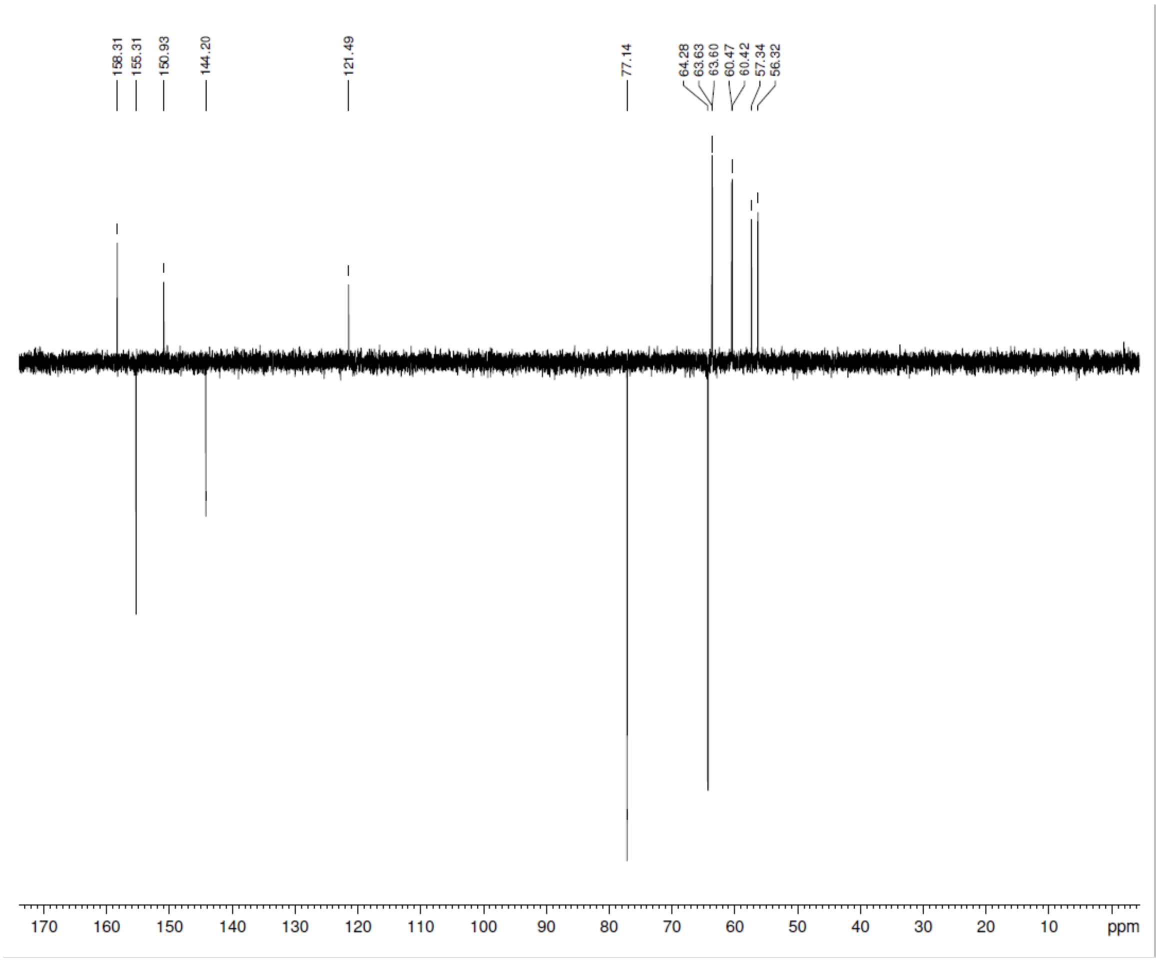
158.31
155.31
150.93
144.20
121.49
77.14
64.28
63.63
63.60
60.47
60.42
57.34
56.32
170 160 150 140 130 120 110 100 90 80 70 60 50 40 30 20 10 ppm

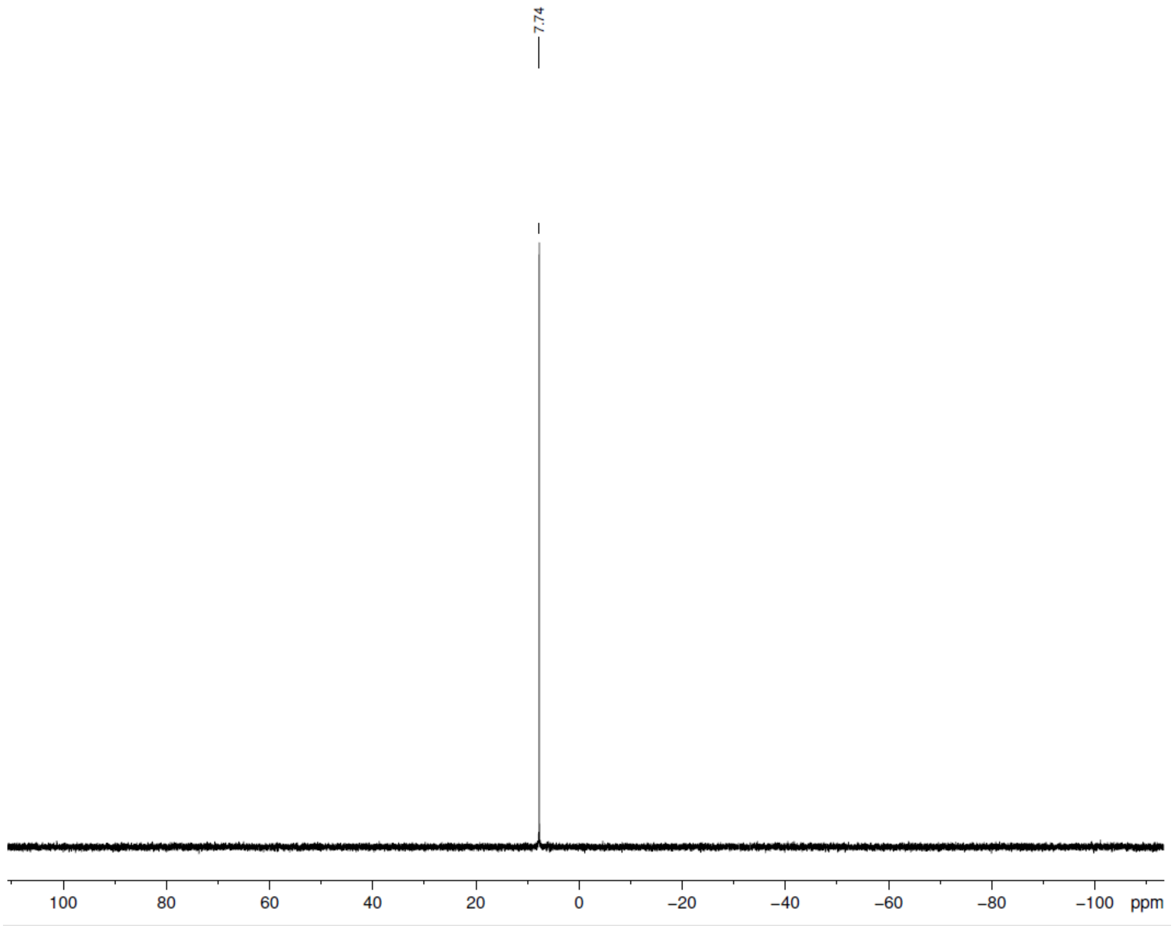
7.74
100 80 60 40 20 0 −20 −40 −60 −80 −100 ppm

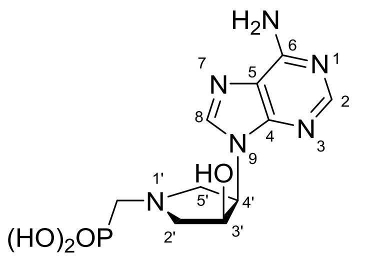

10

^{1}H NMR (499.8 MHz, D_2O, T = 25 °C, pD = 6.7): 3.33 (dd, 1H, J_{gem} = 14.1, $J_{H,P}$ = 11.2, $CH_a\mathbf{H_b}P$); 3.37 (dd, 1H, J_{gem} = 14.1, $J_{H,P}$ = 11.6, $C\mathbf{H_a}H_bP$); 3.83 (dd, 1H, J_{gem} = 13.0, $J_{2'',3'}$ = 6.1, H-2"); 3.87 (dd, 1H, J_{gem} = 13.0, $J_{2',3'}$ = 3.5, H-2'); 4.01 (dd, 1H, J_{gem} = 12.8, $J_{5'',4'}$ = 7.9, H-5''); 4.30 (dd, 1H, J_{gem} = 12.8, $J_{5',4'}$ = 5.0, H-5'); 4.25); 4.92 (td, 1H, $J_{3',4'}$ = $J_{3',2''}$ = 6.1, $J_{3',2'}$ = 3.5, H-3'); 5.47 (ddd, 1H, $J_{4',5''}$ = 7.9, $J_{4',3'}$ = 6.2, $J_{4',5'}$ = 5.0, H-4'); 8.23 (s, 1H, H-8); 8.27 (s, 1H, H-2).

^{13}C NMR (125.7 MHz, D_2O, T = 25 °C, pD = 6.7): 56.73 (d, $J_{C,P}$ = 127.6, CH_2P); 59.20 (C-4'); 59.71 (d, $J_{C,P}$ = 5.8, C-5'); 63.92 (d, $J_{C,P}$ = 4.0, C-2'); 71.51 (C-3'); 121.04 (C-5); 145.04 (C-8); 151.71 (C-4); 155.31 (C-2); 158.35 (C-6).

^{31}P{1H} NMR (202.3 MHz, D_2O, T = 25 °C, pD = 6.7): 7.24.

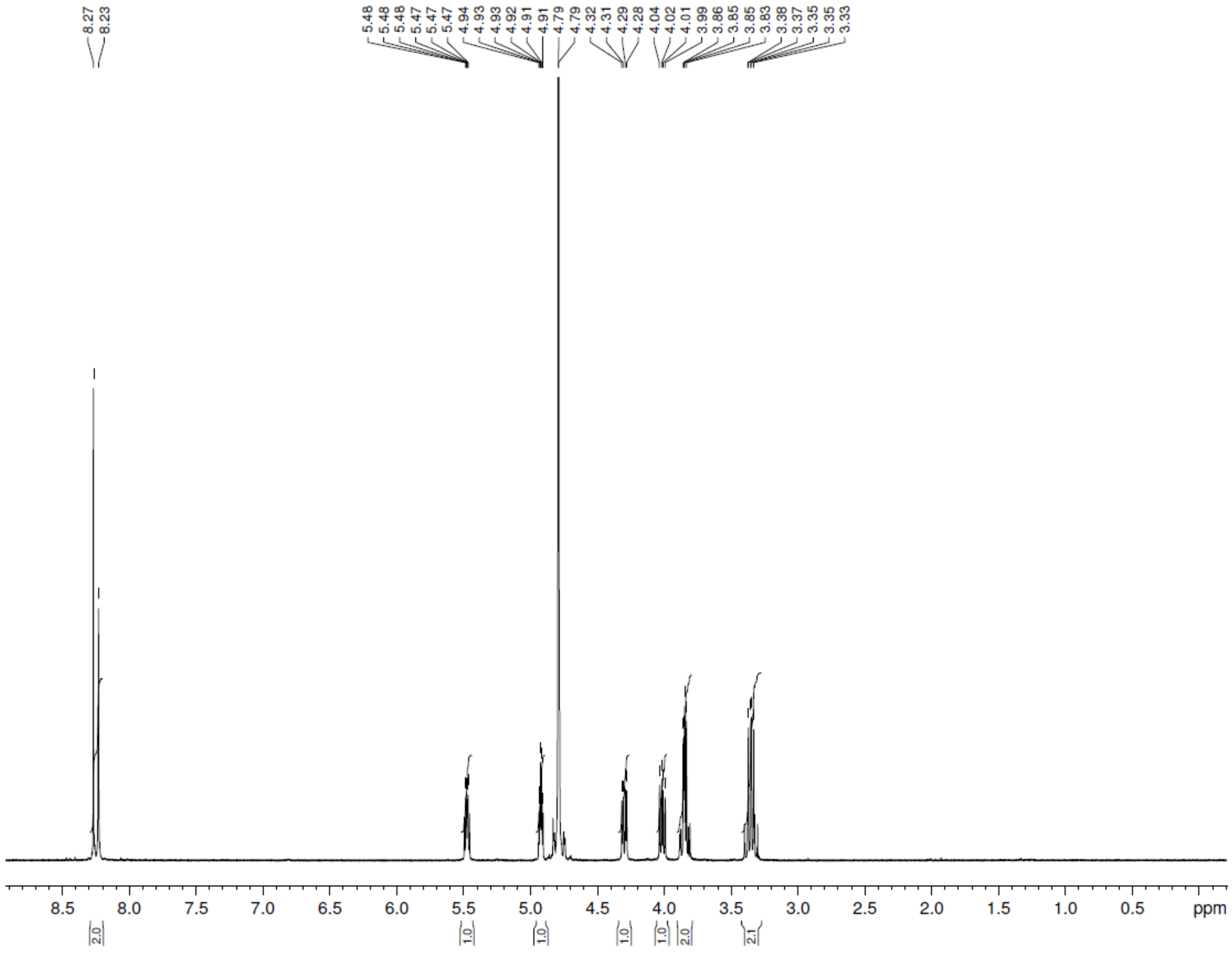

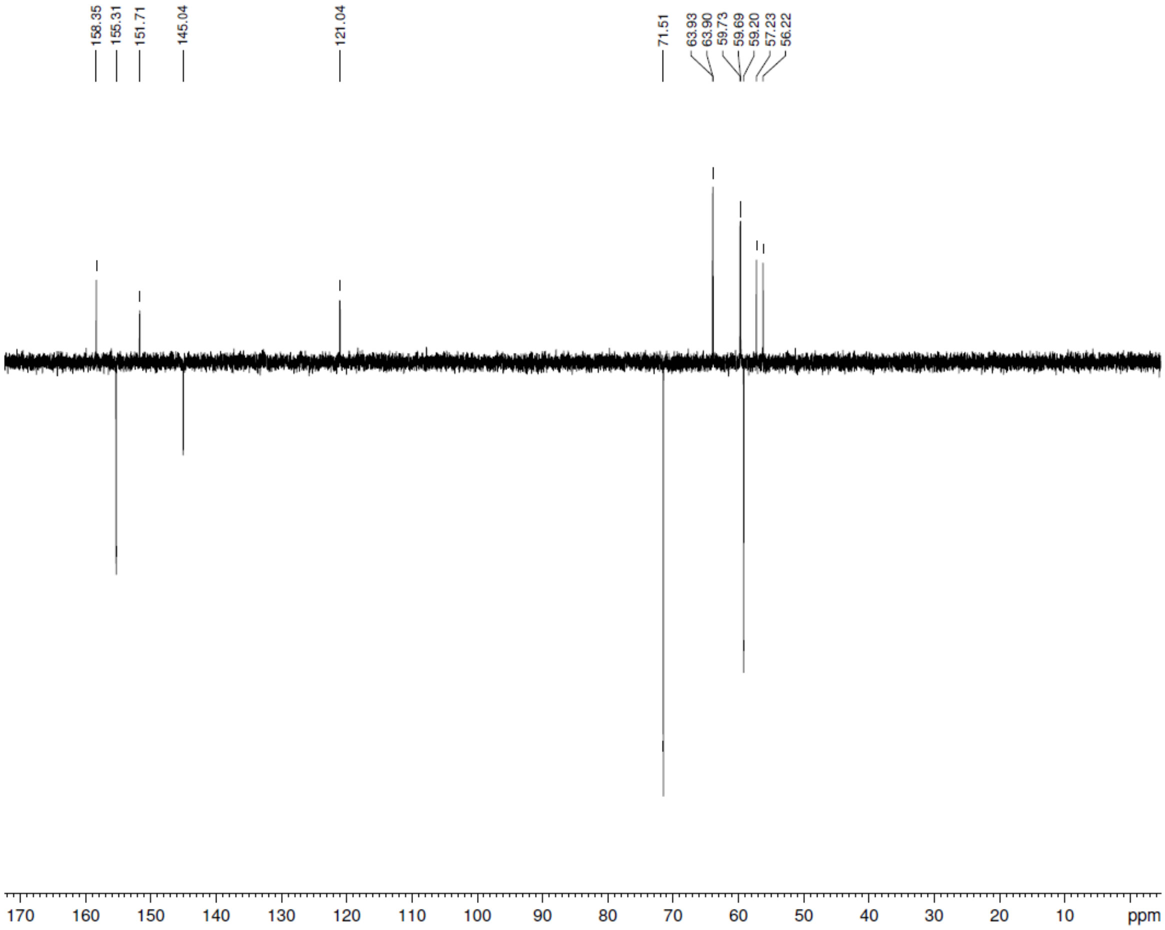
158.35
155.31
151.71
145.04
121.04
71.51
63.93
63.90
59.73
59.69
59.20
57.23
56.22
170 160 150 140 130 120 110 100 90 80 70 60 50 40 30 20 10 ppm

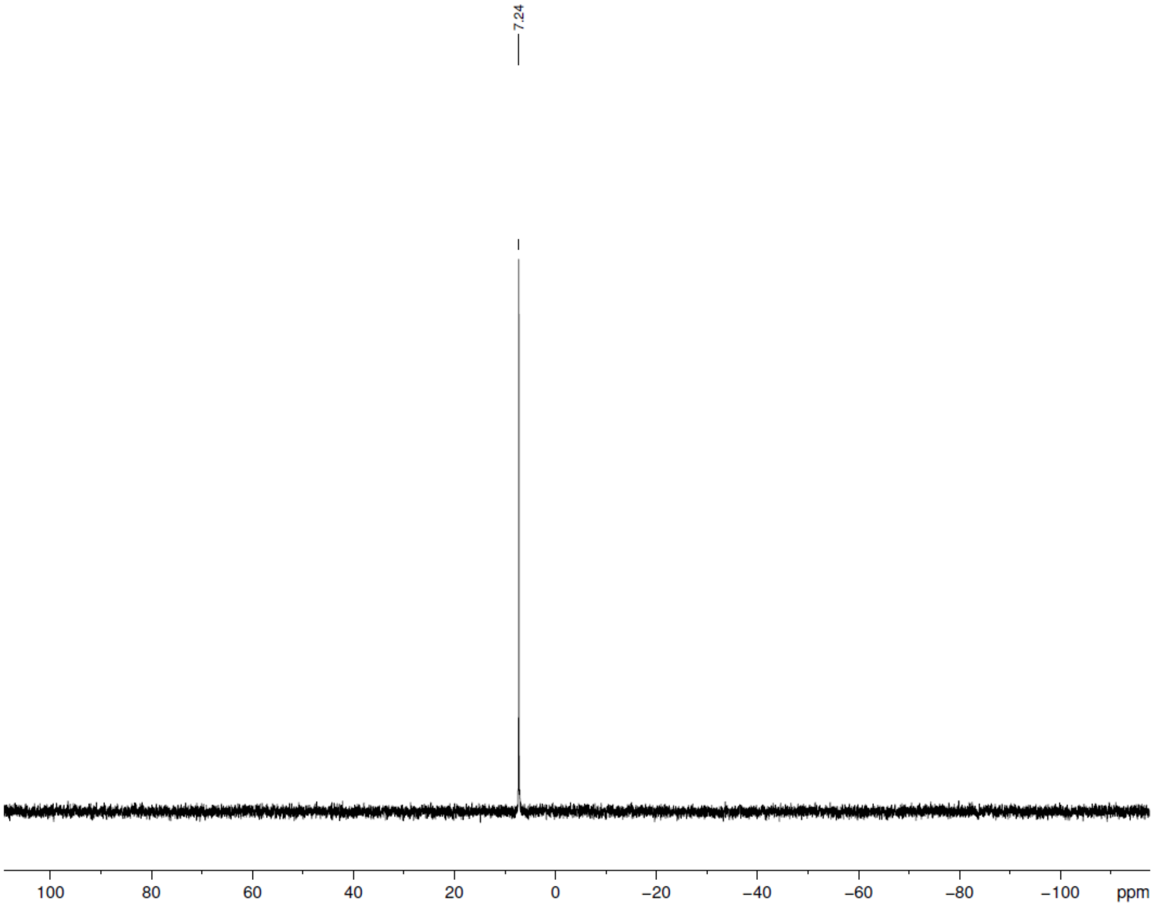
7.24
100 80 60 40 20 0 −20 −40 −60 −80 −100 ppm

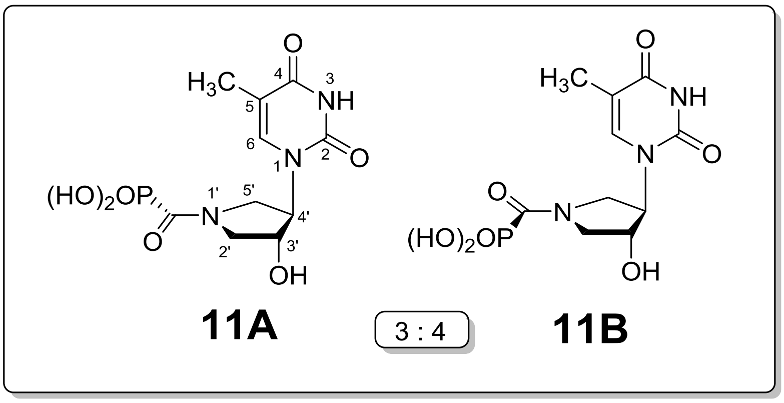

Rotamer A:

^{1}H NMR (400.1 MHz, D_2O, T = 25 °C, pD = 6.4): 1.88 (d, 3H, ^{4}J = 1.2, CH_3); 3.47 (ddd, 1H, J_{gem} = 13.6, $J_{2'',3'}$ = 4.8, $J_{H,P}$ = 1.7, H-2''); 3.85 (ddd, 1H, J_{gem} = 13.6, $J_{2',3'}$ = 6.8, $J_{H,P}$ = 1.6, H-2'); 4.12 (dd, 1H, J_{gem} = 12.9, $J_{5',4'}$ = 6.2, H-5'); 4.41 (dd, 1H, J_{gem} = 12.9, $J_{5'',4'}$ = 7.7, H-5''); 4.59 (ddd, 1H, $J_{3',2'}$ = 6.8, $J_{3',4'}$ = 5.4, $J_{3',2''}$ = 4.8, H-3'); 4.91 (ddd, 1H, $J_{4',5''}$ = 7.7, $J_{4',5'}$ = 6.2, $J_{4',3'}$ = 5.4, H-4'); 7.43 (q, 1H, ^{4}J = 1.2, H-6).

^{13}C NMR (100.6 MHz, D_2O, T = 25 °C, pD = 6.4): 14.24 (CH_3); 50.26 (C-5'); 52.88 (d, $J_{C,P}$ = 4.7, C-2'); 64.31 (C-4'); 73.07 (C-3'); 114.39 (C-5); 141.28 (C-6); 155.03 (C-2); 169.15 (C-4); 177.87 (d, $J_{C,P}$ = 202.7, P-CO).

^{31}P{1H} NMR (162.0 MHz, D_2O, T = 25 °C, pD = 6.4): -2.72.

Rotamer B:

^{1}H NMR (400.1 MHz, D_2O, T = 25 °C, pD = 6.4): 1.88 (d, 3H, ^{4}J = 1.2, CH_3); 3.68 (ddd, 1H, J_{gem} = 13.6, $J_{5',4'}$ = 6.1, $J_{H,P}$ = 1.6, H-5'); 3.81 (dd, 1H, J_{gem} = 12.3, $J_{2'',3'}$ = 5.7, H-2''); 4.00 (ddd, 1H, J_{gem} = 13.6, $J_{5'',4'}$ = 8.5, $J_{H,P}$ = 1.6, H-5''); 4.29 (dd, 1H, J_{gem} = 12.3, $J_{2',3'}$ = 6.1, H-2'); 4.60 (ddd, 1H, $J_{3',2'}$ = 6.1, $J_{3',2''}$ = 5.7, $J_{3',4'}$ = 5.5, H-3'); 4.94 (ddd, 1H, $J_{4',5''}$ = 8.5, $J_{4',5'}$ = 6.1, $J_{4',3'}$ = 5.5, H-4'); 7.42 (q, 1H, ^{4}J = 1.2, H-6).

^{13}C NMR (100.6 MHz, D_2O, T = 25 °C, pD = 6.4): 14.17 (CH_3); 48.81 (d, $J_{C,P}$ = 5.3, C-5'); 54.44 (C-2'); 62.37 (C-4'); 74.64 (C-3'); 114.45 (C-5); 141.35 (C-6); 155.08 (C-2); 169.14 (C-4); 177.87 (d, $J_{C,P}$ = 202.7, P-CO).

^{31}P{1H} NMR (162.0 MHz, D_2O, T = 25 °C, pD = 6.4): -2.63.

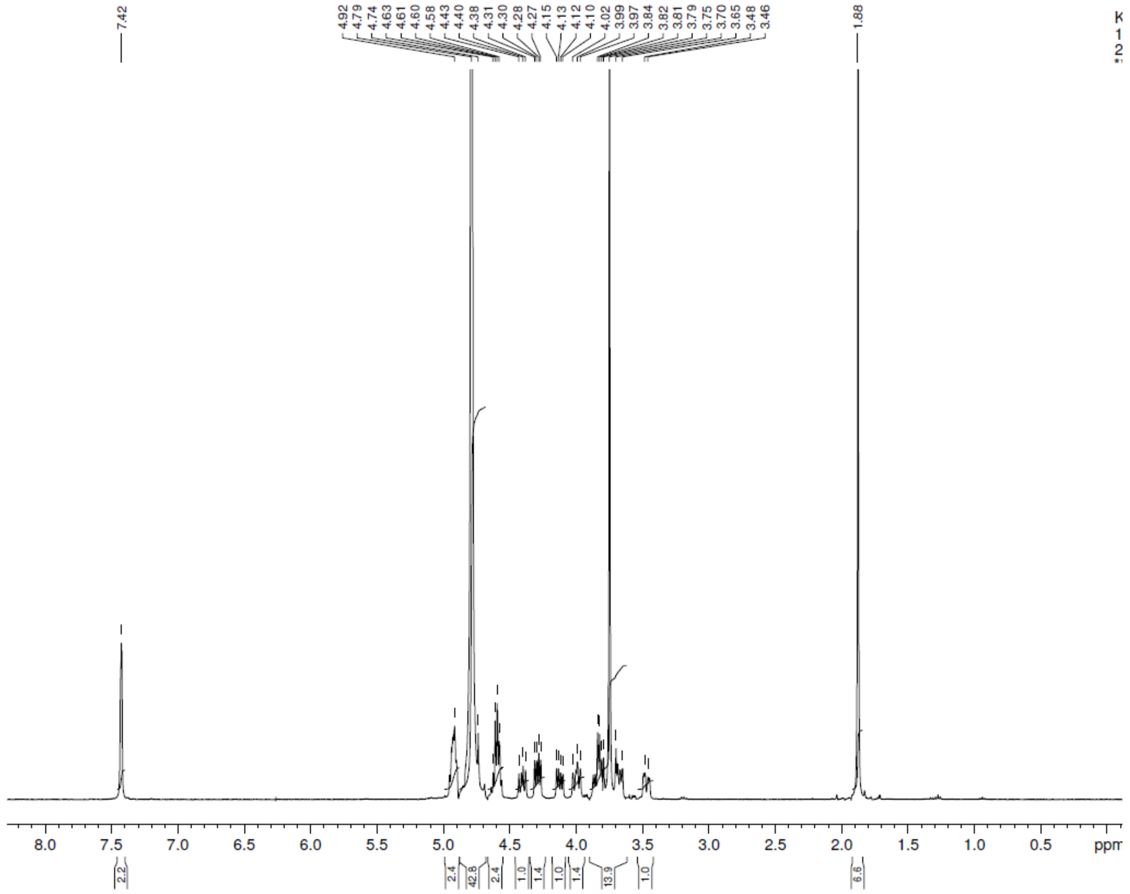
7.42
4.92 4.79 4.74 4.63 4.61 4.60 4.58 4.43 4.40 4.38 4.31 4.30 4.28 4.27 4.15 4.13 4.12 4.10 4.02 3.99 3.97 3.84 3.82 3.81 3.79 3.75 3.70 3.65 3.48 3.46
1.88
8.0 7.5 7.0 6.5 6.0 5.5 5.0 4.5 4.0 3.5 3.0 2.5 2.0 1.5 1.0 0.5 ppm
2.2
2.4 42.8 2.4 1.0 1.4 1.0 1.4 13.9 1.0
6.6

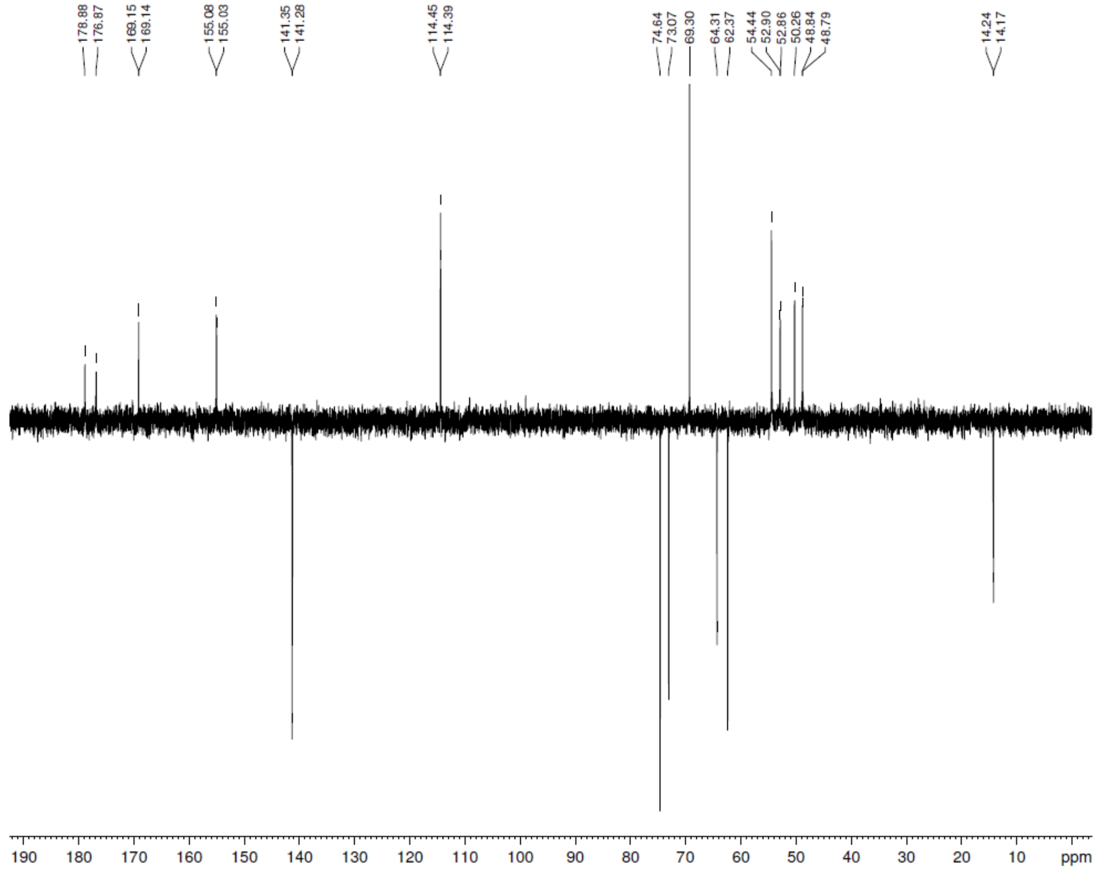
178.88 176.87 169.15 169.14 155.08 155.03 141.35 141.28 114.45 114.39 74.64 73.07 69.30 64.31 62.37 54.44 52.90 52.86 50.26 48.84 48.79 14.24 14.17
190 180 170 160 150 140 130 120 110 100 90 80 70 60 50 40 30 20 10 ppm

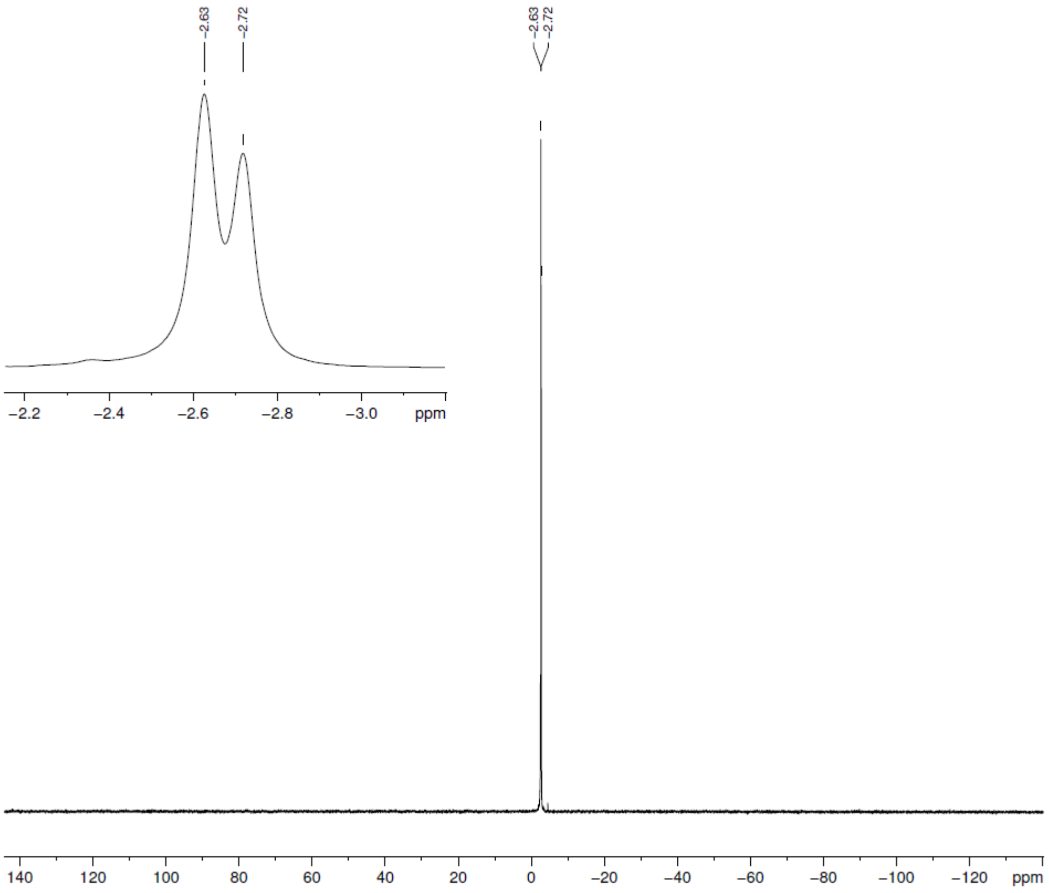
−2.63
−2.72
−2.2
−2.4
−2.6
−2.8
−3.0
ppm
−2.63
−2.72
140
120
100
80
60
40
20
0
−20
−40
−60
−80
−100
−120
ppm

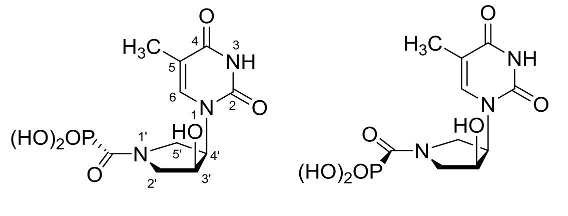

12A 10 : 11 **12B**

Rotamer A:

^{1}H NMR (600.1 MHz, D_2O, T = 25 °C, pD = 5.9): 1.891 (d, 3H, ^4J = 1.2, CH_3); 3.57 (dt, 1H, J_{gem} = 13.7, $J_{2',3'}$ = $J_{H,P}$ = 2.1, H-2'); 3.79 (ddd, 1H, J_{gem} = 13.7, $J_{2'',3'}$ = 5.2, $J_{H,P}$ = 2.0, H-2"); 4.17 (dd, 1H, J_{gem} = 11.7, $J_{5',4'}$ = 9.5, H-5'); 4.42 (dd, 1H, J_{gem} = 11.7, $J_{5'',4'}$ = 8.1, H-5"); 4.58 (ddd, 1H, $J_{3',2''}$ = 5.2, $J_{3',4'}$ = 4.5, $J_{3',2'}$ = 2.1, H-3'); 5.09 (ddd, 1H, $J_{4',5'}$ = 9.5, $J_{4',5''}$ = 8.1, $J_{4',3'}$ = 4.5, H-4'); 7.60 (q, 1H, ^4J = 1.2, H-6).

^{13}C NMR (150.9 MHz, D_2O, T = 25 °C, pD = 5.9): 14.22 (CH_3); 48.94 (C-5'); 54.51 (d, $J_{C,P}$ = 4.5, C-2'); 59.37 (C-4'); 70.15 (C-3'); 113.06 (C-5); 142.98 (C-6); 155.29 (C-2); 169.16 (C-4); 178.86 (d, $J_{C,P}$ = 198.0, P-CO).

^{31}P{1H} NMR (202.3 MHz, D_2O, T = 25 °C, pD = 5.9): -2.16.

Rotamer B:

^{1}H NMR (600.1 MHz, D_2O, T = 25 °C, pD = 5.9): 1.887 (d, 3H, ^4J = 1.2, CH_3); 3.80 (ddd, 1H, J_{gem} = 13.0, $J_{5',4'}$ = 8.5, $J_{H,P}$ = 2.1, H-5'); 3.94 (ddd, 1H, J_{gem} = 13.0, $J_{5'',4'}$ = 8.5, $J_{H,P}$ = 2.0, H-5"); 4.05 (dd, 1H, J_{gem} = 12.7, $J_{2',3'}$ = 3.3, H-2'); 4.12 (dd, 1H, J_{gem} = 12.7, $J_{2'',3'}$ = 4.8, H-2"); 4.59 (ddd, 1H, $J_{3',2''}$ = 4.8, $J_{3',4'}$ = 4.6, $J_{3',2'}$ = 3.3, H-3'); 5.18 (dt, 1H, $J_{4',5'}$ = 8.5, $J_{4',5''}$ = 8.5, $J_{4',3'}$ = 4.6, H-4'); 7.57 (q, 1H, ^4J = 1.2, H-6).

^{13}C NMR (100.6 MHz, D_2O, T = 25 °C, pD = 5.9): 14.18 (CH_3); 48.86 (d, $J_{C,P}$ = 5.1, C-5'); 55.89 (C-2'); 57.63 (C-4'); 72.22 (C-3'); 113.14 (C-5); 142.86 (C-6); 155.37 (C-2); 169.16 (C-4); 178.88 (d, $J_{C,P}$ = 198.5, P-CO).

^{31}P{1H} NMR (162.0 MHz, D_2O, T = 25 °C, pD = 5.9): -2.28.

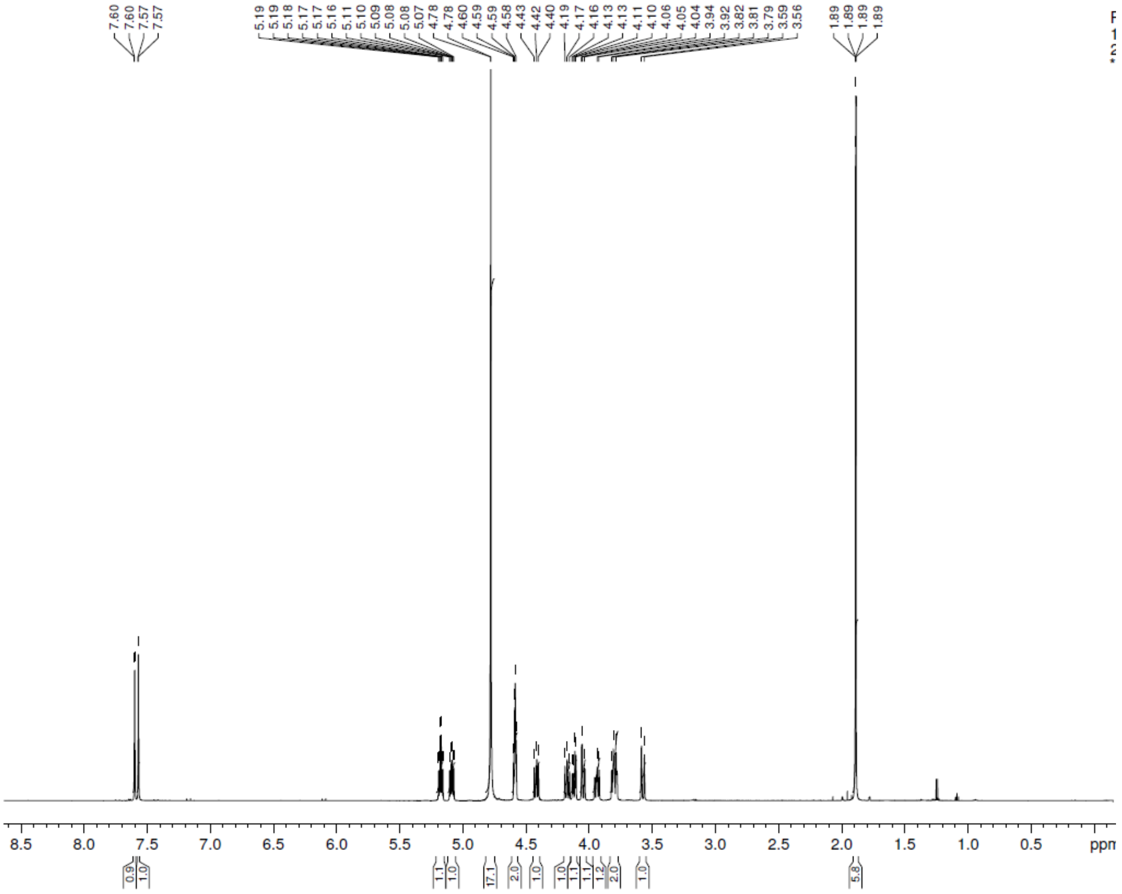

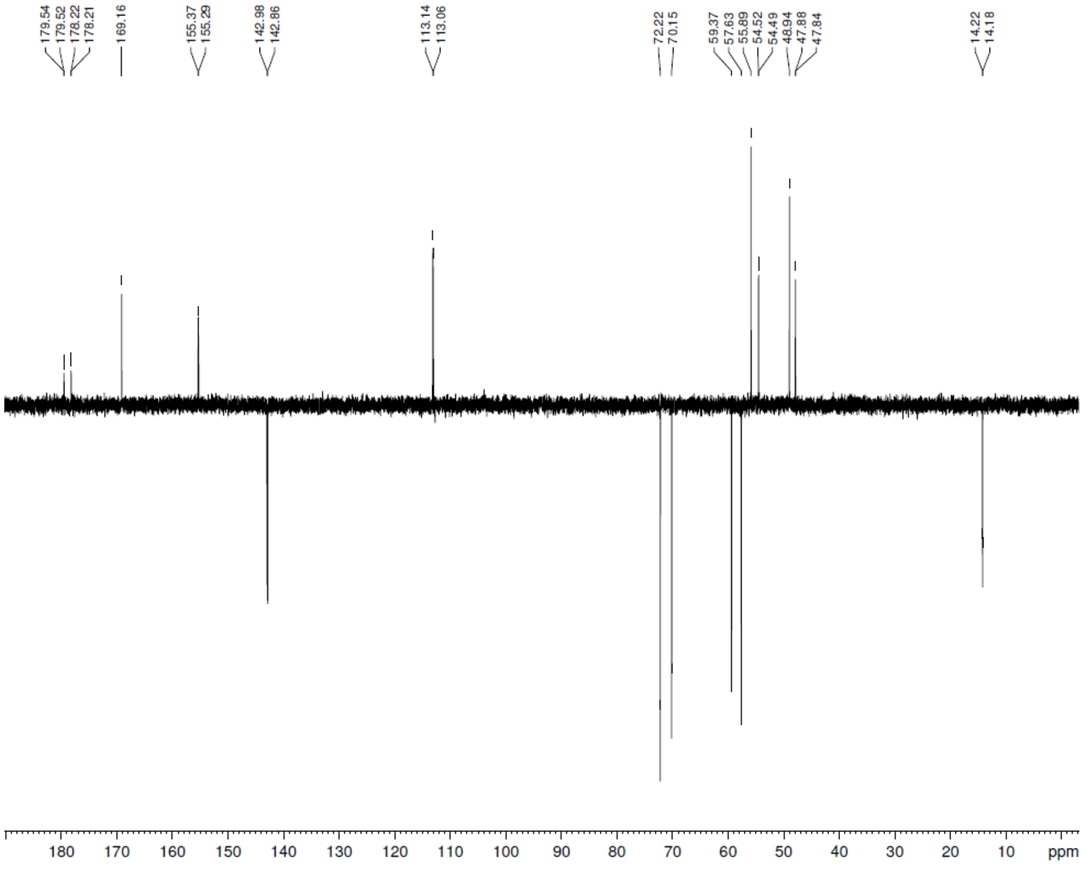

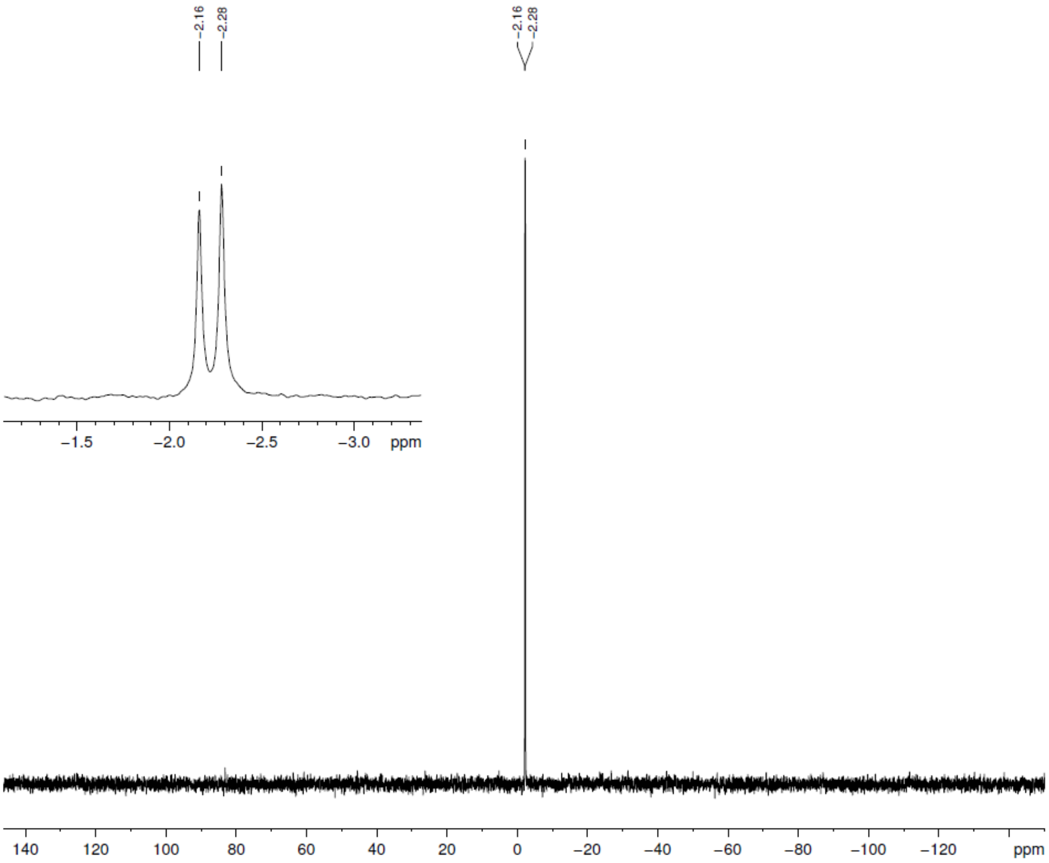

−2.16
−2.28
−1.5
−2.0
−2.5
−3.0
ppm
140
120
100
80
60
40
20
0
−20
−40
−60
−80
−100
−120
ppm

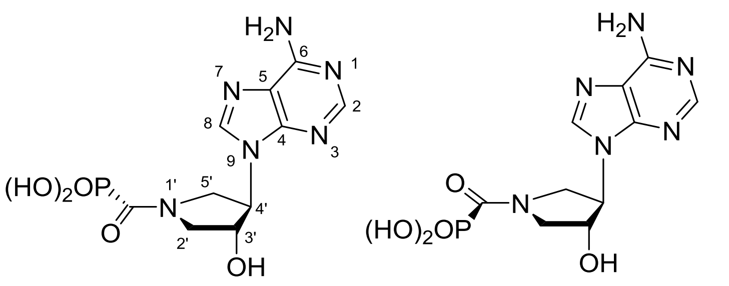

13A 10 : 11 **13B**

Rotamer A:

^{1}H NMR (600.1 MHz, D_2O, T = 25 °C, pD = 6.1): 3.54 (dddd, 1H, J_{gem} = 13.6, $J_{2'',3'}$ = 4.1, $J_{H,P}$ = 1.9, $J_{2'',5''}$ = 0.9, H-2"); 3.83 (ddd, 1H, J_{gem} = 13.6, $J_{2',3'}$ = 6.1, $J_{H,P}$ = 1.9, H-2'); 4.55 (ddd, 1H, J_{gem} = 13.0, $J_{5',4'}$ = 5.6, $J_{5',3'}$ = 0.9, H-5'); 4.57 (ddd, 1H, J_{gem} = 13.0, $J_{5'',4'}$ = 6.3, $J_{5'',2''}$ = 0.9, H-5"); 4.73 (dddd, 1H, $J_{3',2'}$ = 6.1, $J_{3',4'}$ = 4.5, $J_{3',2''}$ = 4.1, $J_{3',5'}$ = 0.9, H-3'); 5.01 (ddd, 1H, $J_{4',5''}$ = 6.3, $J_{4',5'}$ = 5.6, $J_{4',3'}$ = 4.5, H-4'); 8.134 (s, 1H, H-8); 8.151 (s, 1H, H-2).

^{13}C NMR (150.9 MHz, D_2O, T = 25 °C, pD = 6.1): 51.65 (C-5'); 53.11 (d, $J_{C,P}$ = 4.6, C-2'); 62.87 (C-4'); 73.75 (C-3'); 121.29 (C-5); 142.56 (C-8); 151.58 (C-4); 154.92 (C-2); 157.98 (C-6); 179.15 (d, $J_{C,P}$ = 197.5, P-CO).

^{31}P{1H} NMR (202.3 MHz, D_2O, T = 25 °C, pD = 6.1): -2.25.

Rotamer B:

^{1}H NMR (600.1 MHz, D_2O, T = 25 °C, pD = 6.1): 3.95 (dd, 1H, J_{gem} = 12.6, $J_{2'',3'}$ = 5.2, H-2"); 4.01 (ddd, 1H, J_{gem} = 13.6, $J_{5',4'}$ = 5.5, $J_{H,P}$ = 1.9, H-5'); 4.19 (ddd, 1H, J_{gem} = 13.6, $J_{5'',4'}$ = 7.8, $J_{H,P}$ = 2.0, H-5"); 4.31 (ddd, 1H, J_{gem} = 12.6, $J_{2',3'}$ = 5.8, $J_{2',5'}$ = 0.8, H-2'); 4.76 (ddd, 1H, $J_{3',2'}$ = 5.8, $J_{3',4'}$ = 5.3, $J_{3',2''}$ = 5.2, H-3'); 4.99 (ddd, 1H, $J_{4',5''}$ = 7.8, $J_{4',5'}$ = 5.5, $J_{4',3'}$ = 5.3, H-4'); 8.130 (s, 1H, H-8); 8.149 (s, 1H, H-2).

^{13}C NMR (150.9 MHz, D_2O, T = 25 °C, pD = 6.1): 50.23 (d, $J_{C,P}$ = 4.9, C-5'); 54.64 (C-2'); 60.88 (C-4'); 75.44 (C-3'); 121.29 (C-5); 142.73 (C-8); 151.62 (C-4); 154.95 (C-2); 158.00 (C-6); 179.24 (d, $J_{C,P}$ = 197.0, P-CO).

^{31}P{1H} NMR (202.3 MHz, D_2O, T = 25 °C, pD = 6.1): -2.23.

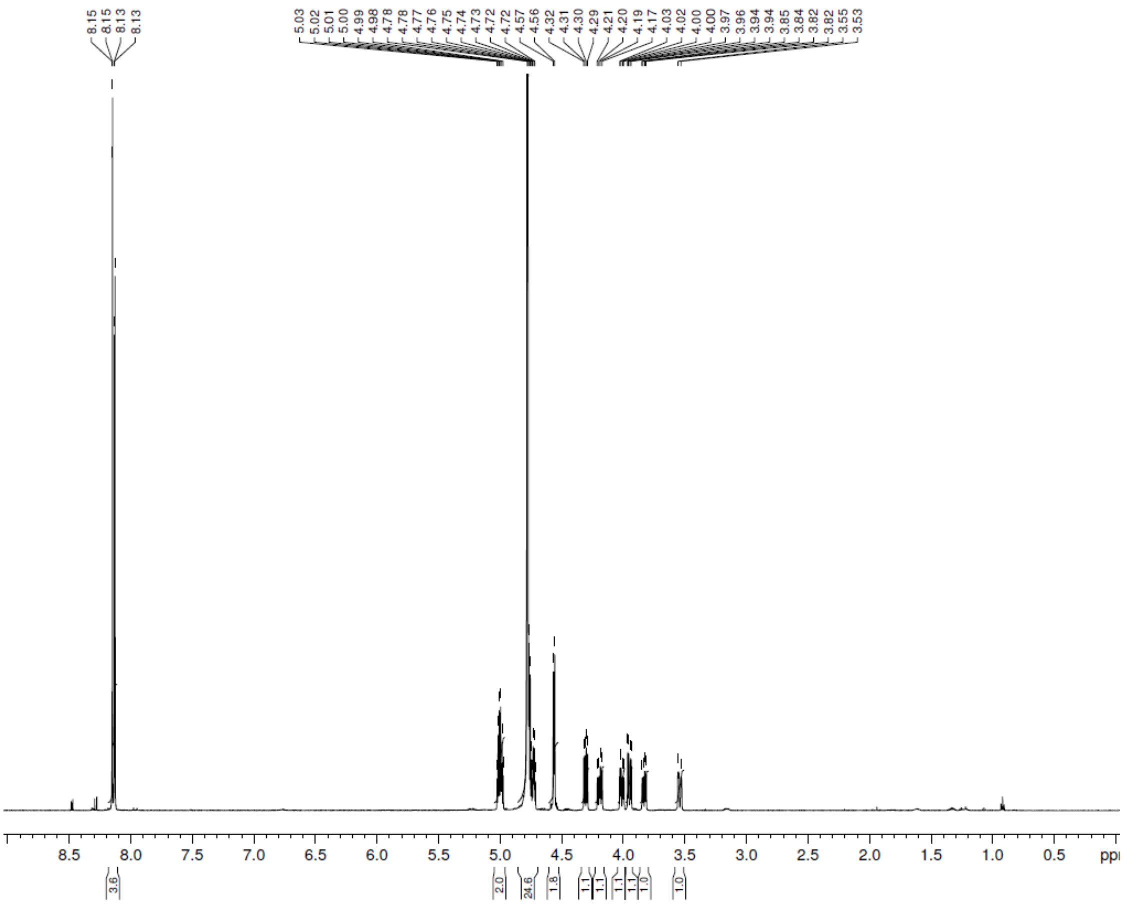
8.15
8.15
8.13
8.13
5.03
5.02
5.01
5.00
4.99
4.98
4.78
4.77
4.76
4.75
4.74
4.73
4.72
4.72
4.57
4.56
4.32
4.31
4.30
4.29
4.21
4.20
4.19
4.17
4.03
4.02
4.00
4.00
3.97
3.96
3.94
3.94
3.85
3.84
3.82
3.82
3.55
3.53
8.5
8.0
7.5
7.0
6.5
6.0
5.5
5.0
4.5
4.0
3.5
3.0
2.5
2.0
1.5
1.0
0.5
ppm
3.6
2.0
24.6
1.8
1.1
1.1
1.1
1.1
1.0
1.0

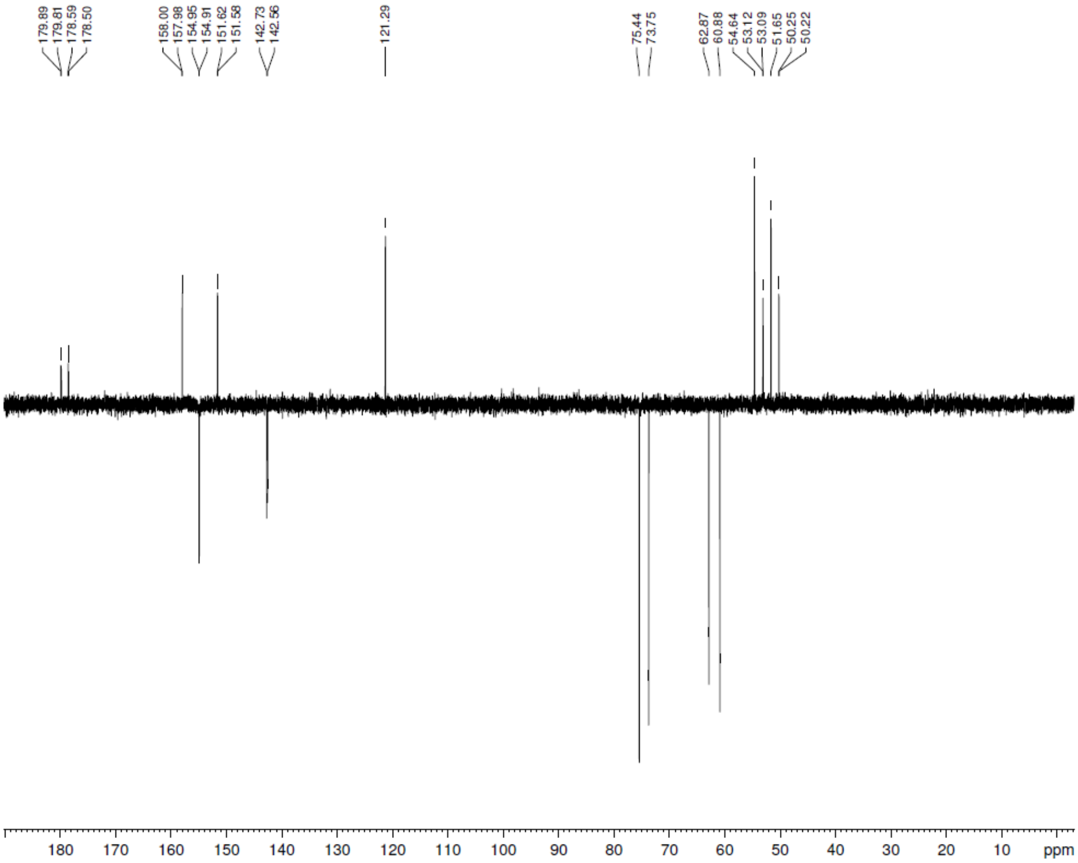
179.89
179.81
178.59
178.50
158.00
157.98
154.95
154.91
151.62
151.58
142.73
142.56
121.29
75.44
73.75
62.87
60.88
54.64
53.12
53.09
51.65
50.25
50.22
180
170
160
150
140
130
120
110
100
90
80
70
60
50
40
30
20
10
ppm

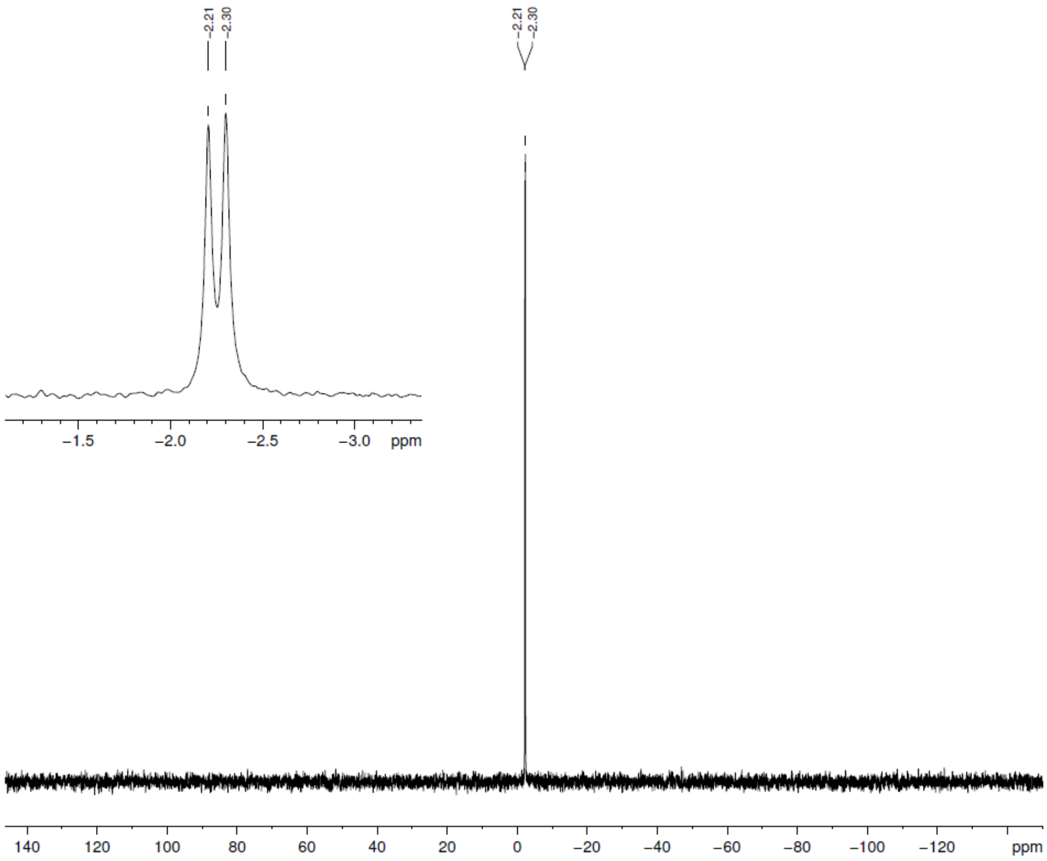

−2.21
−2.30
−1.5
−2.0
−2.5
−3.0
ppm
−2.21
−2.30
140
120
100
80
60
40
20
0
−20
−40
−60
−80
−100
−120
ppm

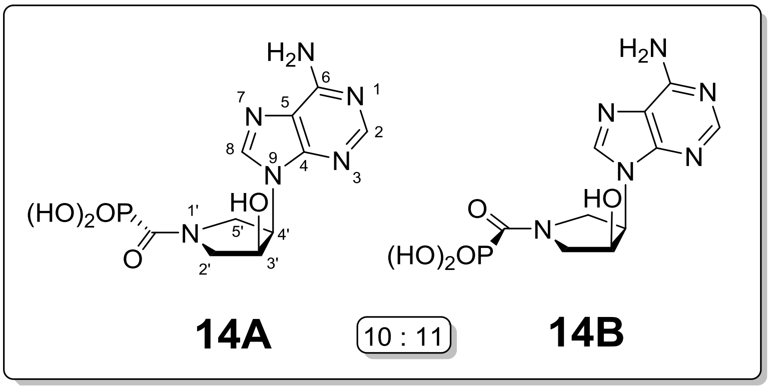

Rotamer A:

^{1}H NMR (600.1 MHz, D_2O, T = 25 °C, pD = 5.9): 3.65 (dt, 1H, J_{gem} = 13.7, $J_{2',3'}$ = $J_{H,P}$ = 2.1, H-2'); 3.90 (ddd, 1H, J_{gem} = 13.7, $J_{2'',3'}$ = 5.1, $J_{H,P}$ = 1.9, H-2"); 4.41 (dd, 1H, J_{gem} = 11.6, $J_{5',4'}$ = 9.2, H-5'); 4.66 (dd, 1H, J_{gem} = 11.6, $J_{5'',4'}$ = 7.6, H-5"); 4.693 (ddd, 1H, $J_{3',2''}$ = 5.1, $J_{3',4'}$ = 4.2, $J_{3',2'}$ = 2.1, H-3'); 5.19 (ddd, 1H, $J_{4',5'}$ = 9.2, $J_{4',5''}$ = 7.6, $J_{4',3'}$ = 4.2, H-4'); 8.158 (s, 1H, H-2); 8.262 (s, 1H, H-8).

^{13}C NMR (150.9 MHz, D_2O, T = 25 °C, pD = 5.9): 50.18 (C-5'); 54.30 (d, $J_{C,P}$ = 4.5, C-2'); 58.95 (C-4'); 70.35 (C-3'); 120.80 (C-5); 143.65 (C-8); 151.93 (C-4); 154.89 (C-2); 157.95 (C-6); 178.88 (d, $J_{C,P}$ = 198.3, P-CO).

^{31}P{1H} NMR (202.3 MHz, D_2O, T = 25 °C, pD = 5.9): -2.21.

Rotamer B:

^{1}H NMR (600.1 MHz, D_2O, T = 25 °C, pD = 5.9): 4.04 (ddd, 1H, J_{gem} = 12.8, $J_{5',4'}$ = 8.3, $J_{H,P}$ = 1.9, H-5'); 4.14 (dd, 1H, J_{gem} = 13.0, $J_{2',3'}$ = 3.5, H-2'); 4.18 (ddd, 1H, J_{gem} = 12.8, $J_{5'',4'}$ = 8.2, $J_{H,P}$ = 1.8, H-5"); 4.20 (dd, 1H, J_{gem} = 12.6, $J_{2'',3'}$ = 4.6, H-2"); 4.698 (ddd, 1H, $J_{3',2''}$ = 4.6, $J_{3',4'}$ = 4.3, $J_{3',2'}$ = 3.5, H-3'); 5.21 (ddd, 1H, $J_{4',5'}$ = 8.3, $J_{4',5''}$ = 8.2, $J_{4',3'}$ = 4.3, H-4'); 8.163 (s, 1H, H-2); 8.261 (s, 1H, H-8).

^{13}C NMR (150.9 MHz, D_2O, T = 25 °C, pD = 5.9): 49.06 (d, $J_{C,P}$ = 5.2, C-5'); 55.63 (C-2'); 57.39 (C-4'); 72.34 (C-3'); 120.82 (C-5); 143.66 (C-8); 152.04 (C-4); 154.90 (C-2); 157.95 (C-6); 178.91 (d, $J_{C,P}$ = 197.9, P-CO).

^{31}P{1H} NMR (202.3 MHz, D_2O, T = 25 °C, pD = 5.9): -2.30.

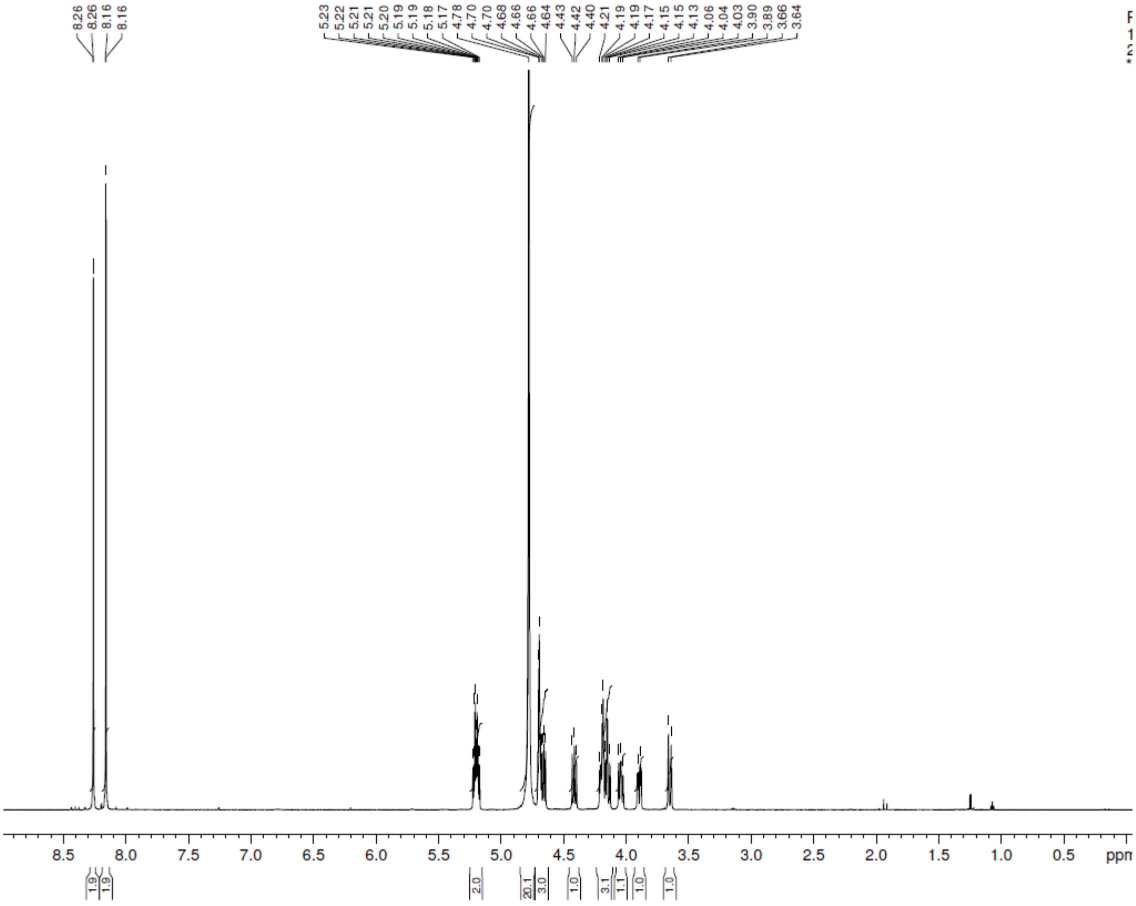
8.26
8.26
8.16
8.16
5.23
5.22
5.21
5.21
5.20
5.19
5.19
5.18
5.17
4.78
4.70
4.70
4.68
4.66
4.66
4.64
4.43
4.42
4.40
4.21
4.19
4.19
4.17
4.15
4.15
4.13
4.06
4.04
4.03
3.90
3.89
3.66
3.64
1.9
1.9
2.0
20.1
3.0
1.0
3.1
1.1
1.0
1.0
ppm

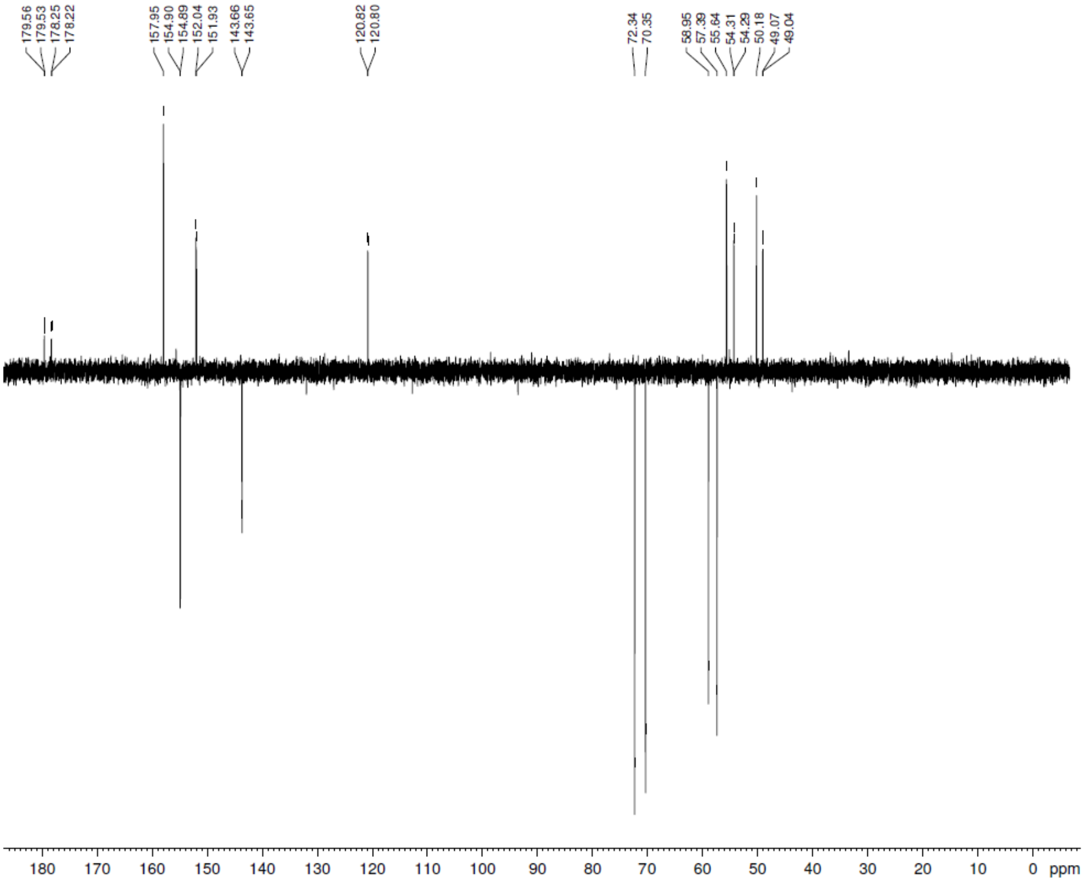
179.56
179.53
178.25
178.22
157.95
154.90
154.89
152.04
151.93
143.66
143.65
120.82
120.80
72.34
70.35
58.95
57.39
55.64
54.31
54.29
50.18
49.07
49.04
ppm

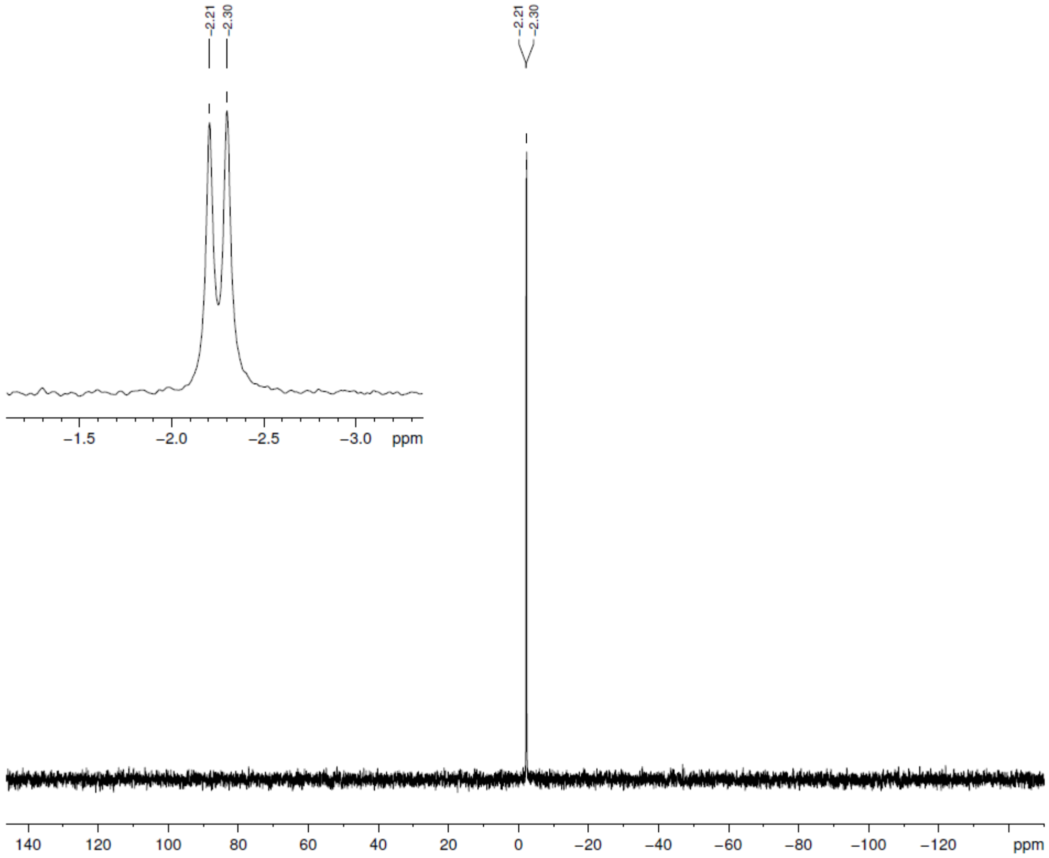

-2.21
-2.30
-1.5
-2.0
-2.5
-3.0
ppm
-2.21
-2.30
140
120
100
80
60
40
20
0
-20
-40
-60
-80
-100
-120
ppm
